# What do we want to get out of this? a critical interpretive synthesis of the value of process evaluations, with a practical planning framework

**DOI:** 10.1186/s12874-022-01767-7

**Published:** 2022-11-25

**Authors:** Caroline French, Anna Dowrick, Nina Fudge, Hilary Pinnock, Stephanie J. C. Taylor

**Affiliations:** 1 Wolfson Institute of Population Health, Barts and The London School of Medicine and Dentistry, Queen Mary University of London, Yvonne Carter Building, 58 Turner Street, London, E1 2AB UK; 2 Nuffield Department of Primary Care Health Sciences, University of Oxford, Radcliffe Primary Care Building, Radcliffe Observatory Quarter, Woodstock Road, Oxford, OX2 6GC UK; 3 Usher Institute, The University of Edinburgh, Doorway 3, Medical School, Teviot Place, Edinburgh, EH8 9AG UK

**Keywords:** Process evaluation, Research impact, Values, Systematic review, Critical interpretive synthesis

## Abstract

**Background:**

Process evaluations aim to understand how complex interventions bring about outcomes by examining intervention mechanisms, implementation, and context. While much attention has been paid to the methodology of process evaluations in health research, the value of process evaluations has received less critical attention. We aimed to unpack how value is conceptualised in process evaluations by identifying and critically analysing 1) how process evaluations may create value and 2) what kind of value they may create.

**Methods:**

We systematically searched for and identified published literature on process evaluation, including guidance, opinion pieces, primary research, reviews, and discussion of methodological and practical issues**.** We conducted a critical interpretive synthesis and developed a practical planning framework.

**Results:**

We identified and included 147 literature items. From these we determined three ways in which process evaluations may create value or negative consequences: 1) through the socio-technical processes of ‘doing’ the process evaluation, 2) through the features/qualities of process evaluation knowledge, and 3) through using process evaluation knowledge. We identified 15 value themes. We also found that value varies according to the characteristics of individual process evaluations, and is subjective and context dependent.

**Conclusion:**

The concept of value in process evaluations is complex and multi-faceted. Stakeholders in different contexts may have very different expectations of process evaluations and the value that can and should be obtained from them. We propose a planning framework to support an open and transparent process to plan and create value from process evaluations and negotiate trade-offs. This will support the development of joint solutions and, ultimately, generate more value from process evaluations to all.

**Supplementary Information:**

The online version contains supplementary material available at 10.1186/s12874-022-01767-7.

## Background

By examining intervention mechanisms, implementation, and context, process evaluations aim to understand how complex interventions bring about outcomes, shed light on unanticipated effects, and inform optimal integration into existing practice [1]. They are often conducted alongside outcome/effectiveness evaluations of complex interventions, including trials, pilot and feasibility studies, and implementation studies [1]. As recognition has grown that outcome/effectiveness evaluations often provided insufficient understanding of increasingly complex interventions and their effects in different contexts, process evaluations have become increasingly common [1].

Health research funding and commissioning bodies in the UK, including the Medical Research Council [1], National Institute for Health and Care Research [2], and Public Health England (now the UK Health Security Agency) [3], highlight benefits of including process evaluations with evaluations of complex interventions. Their importance is also recognised internationally [4, 5], and in other fields such as education [6]. However, process evaluations have potential disadvantages, including Hawthorne effects [3] and participant burden [7]. There are also possible challenges to conducting process evaluations, including under-resourcing [1], and the complexity of interventions and contexts being evaluated [8].

Questions about how to do process evaluations have been substantially addressed in the literature [1, 9], however to our knowledge the concept of the ‘value’ of process evaluations has not been systematically critically examined. In scoping for this review, we noted that authors often used value-laden but ambiguous adjectives, such as ‘high-quality’, ‘useful’ or ‘necessary’ to describe aspects of process evaluation and process evaluation knowledge, without defining these terms. Some aspects of value have been considered, including whether process evaluations can satisfactorily meet the aim of explaining outcomes [10], the value of pragmatic formative process evaluation [11], and the reported value of process evaluations in pragmatic randomised controlled trials (RCTs) [12]. O’Cathain et al. [13] investigated the value of combining RCTs and qualitative research but did not specifically examine process evaluations.

Recommendations and assertions about value are likely to reflect authors’ ontological and epistemological standpoints [8], and accordingly there are a variety of interpretations of ‘optimal’ process evaluation design and conduct in the literature. For example, the MRC process evaluation guidance [1] outlines ontological and epistemological debates about how aspects of process such as fidelity and intervention mechanisms may be conceptualised and studied. There are also paradigmatic differences in how complex interventions are conceptualised [14], which impact perspectives on what a process evaluation should be and do.

The concept of “value” in research is multifaceted, with diverse definitions such as*”why we do things, what is important, and to whom”* [15]; *“the established collective moral principles and accepted standards of persons or a social group; principles, standards or qualities considered worthwhile or desirable”* [16]; and “*contribution, impact* and *success”* [13]. Research value is also commonly described in terms of impact, and various typologies and frameworks for categorising and assessing research impact have been proposed [17–20]. Value is also often discussed in terms of financial value and reducing waste brought about through inefficient research processes [21, 22].

In this paper we take a broad perspective on value, aiming to examine the different ways in which the ‘value’ of process evaluation is conceptualised and consider how and why perspectives may differ within the field. Essentially, we seek to establish what may be gained from process evaluation and for whom, potential negative consequences of process evaluations, and what is considered to make a ‘good’ or ‘useful’ process evaluation. In agreement with O’Cathain et al.’s [13] rationale for studying the value of qualitative research in RCTs, we believe taking stock of, and critically analysing the value of process evaluation in its broadest sense is important to advance the methodological knowledge base.

We also believe developing a planning framework of process evaluation value provides practical assistance to researchers designing process evaluations. By making explicit at the outset different expectations of value by different stakeholders, potential tensions may be addressed [16]. Given that process evaluation researchers likely need to prioritise which aspects of interventions to examine and may choose from a wide selection of methods and frameworks [1], we suggest it pertinent to address the question ‘what do we want to get out of this process evaluation?’ *before* addressing the question ‘how are we going to do this process evaluation?’.

Our aims were to identify and critically analyse 1) how process evaluations may create value and negative consequences, and 2) what kind of value process evaluations may create.

## Methods

We conducted a critical interpretive synthesis, broadly following the approach outlined by Dixon-Woods et al. [23]. Accordingly, we aimed to synthesise a diverse body of literature to develop a conceptual framework of a concept (value) that has not been consistently defined and operationalised in this context (process evaluation). The critical interpretive synthesis approach is inductive and interpretive, with the body of literature itself used as an object of analysis as well as individual papers, for example by questioning the inherent assumptions behind what is said and not said [23]. Dixon-Woods et al. [23] describe critical interpretive synthesis as an approach to review and not exclusively a method of synthesis, and do not prescribe a step-by-step method of operationalising their approach. Accordingly, we adopted the basic principles of their approach and adapted it to suit this body of literature, the aims of this review, and our available resources.

Since there has been little previous research into the value of process evaluations, we based this review on literature including process evaluation guidance, opinions about process evaluations, and discussion of methodological and practical issues. Thus, we considered what authors were stating about process evaluations and their value in texts such introductions, discussions, opinion pieces, and editorials, as well as any research findings we did locate in the searches.

### Search strategy

We searched for literature on process evaluation, including guidance, opinion pieces, primary research, reviews, and discussion of methodological and practical issues.

We searched the following sources:

1. Reference lists of four major process evaluation frameworks [1, 4, 9, 24]
2. Forward citation searches of the same four process evaluation frameworks using Web of Science and Google Scholar
3. Medline database search for articles with term “process evaluation*” in title; limited to English language
4. Scopus database search for articles with term “process evaluation*” in title; limited to English language; subjects limited to medicine, social sciences, nursing, psychology, health professions, pharmacology, dentistry
5. ETHOS database for PhD theses with term ‘process evaluation’ in the title (excluded in updated search)
6. Literature items not located by the searches but which we knew contained relevant information about process evaluation from our work in this field, such as broader guidance documents about evaluation methods containing sections on process evaluation.

CF originally conducted the search in September 2017 and updated it in January 2021. In the updated search we excluded the ETHOS database search due to time constraints.

### Definition of process evaluation

We used the definition of process evaluation provided in the Medical Research Council’s process evaluation guidance [1] when selecting items for inclusion: *‘a study which aims to understand the functioning of an intervention, by examining implementation, mechanisms of impact, and contextual factors’.* We chose this definition because the MRC’s process evaluation guidance is extensive and widely cited, and we considered its definition comprehensive.

### Screening, inclusion, and exclusion criteria

We did not aim to include every item of relevant literature, rather to systematically search for and select literature most relevant to our aims. For example, literature on mixed-methods research and process evaluation concepts such as fidelity would have been relevant, however we only included those focusing on the overall concept of process evaluation. Although we only searched health-related sources, we did not limit inclusion to the field of health.

### Inclusion criteria

We included published literature (including editorials, letters, commentaries, book chapters, research articles) that met all the following criteria:

1. Used the term ‘process evaluation’ in line with the above definition
2. Discussed process evaluation in any field, providing ‘process evaluation’ met the definition above
3. Discussed process evaluation accompanying any kind of outcome/effectiveness evaluation, intervention development work, or standalone process evaluation

### Exclusion criteria

1. Items in which term ‘process evaluation’ is used to describe an evaluation not meeting the definition in our review
2. Items which only reported process evaluation protocols or findings – these were only included if they also discussed wider process evaluation issues (e.g. methodological, operational)
3. No full-text available online
4. Not in English language

### Results screening

CF screened the titles and abstracts of all results, obtaining full texts where necessary to aid decisions.

### Data analysis and synthesis

We did not conduct quality appraisal of the included literature as we selected diverse items such as editorials, and synthesised whole texts as qualitative data, rather than aggregated research findings.

This review was inductive and we did not start out with a priori concepts or categories about how process evaluations create value or the type of value they create. We kept in mind however the value system of ‘process’, ‘substantive’ and ‘normative’ values outlined by Gradinger et al. [16] to sensitise us to values possibly stemming from 1) the conduct of process evaluation; 2) the impact of process evaluation or 3) the perceived intrinsic worth of process evaluation, respectively. We considered ‘value’ in its broadest possible sense, and examined what authors stated, implied, and discussed about what may result from a process evaluation (both positive and negative), the purposes of process evaluation, and what makes a ‘good’ or ‘useful’ process evaluation.

Following the critical interpretive synthesis approach [23], we also aimed to be critical through questioning the nature of assumptions and proposed solutions relating to process evaluation issues discussed in the literature. This enabled us to examine how authors covering diverse fields and types of process evaluation variously perceived value in different contexts.

**Fig. 1:**
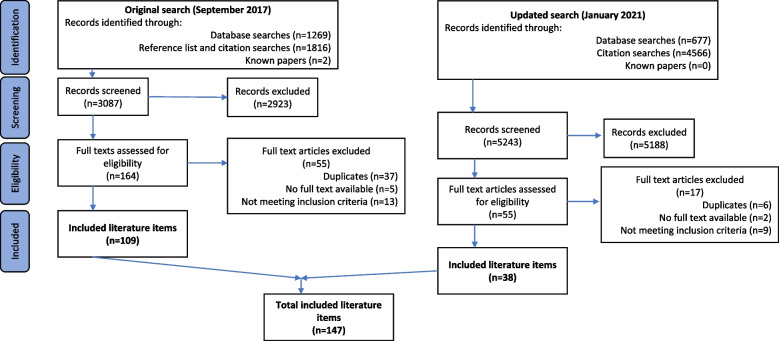
PRISMA flow diagram

CF initially undertook this work as part of her PhD from the original search results in September 2017 with 109 included items (see Fig. 1). Following initial reading of all items to gain familiarity she began the detailed analysis of approximately one third of randomly selected papers (*n* = 40) by extracting sections of text relating to how process evaluations create value and types of value that may be created. She organised these into an initial coding framework, using NVivo to manage the data and noting impressions of the overall literature. She then used this framework to code the remaining items (*n* = 69), amending the framework as necessary. A further 38 literature items were identified following the updated search in January 2021 (see Fig. 1), which CF coded in the same way, further refining the framework.

Dixon-Woods et al. [23] describe the benefits of a multidisciplinary team approach to the whole review and synthesis process. As this paper reports work initiated through individual doctoral work we decided to strengthen and deepen the analysis by independently double coding a total of 36 of the total 147 items (approximately 25%). We used purposive sampling to select the 36 papers for double coding, selecting papers with varied characteristics (year of publication, country of lead author, field of practice, and focus of paper). Four authors coded nine papers each using the coding framework developed by CF, also noting any new themes, interpretations, and areas of disagreement. We brought these to a team discussion to refine the themes and develop the final analysis. We developed this double coding approach as a pragmatic solution to incorporating multiple perspectives into the synthesis, based on our experience of conducting similar narrative reviews and team qualitative data analysis.

From the resulting themes, notes on interpretations, and team discussions we created a narrative and conceptual framework of our analysis, along with a practical planning framework for researchers designing process evaluations.

## Results

### Search results

We included 147 literature items, and our search results are shown in Fig. 1.

Table 1 shows characteristics of the included literature items, with a detailed summary table in additional file 1.

**Table 1:** Characteristics of included literature items

	Number of items (*n* = 147)
**Year of publication**	
2020–2021	12
2015–2019	57
2010–2014	36
2005–2009	21
2000–2004	15
Pre-2000	6
**Type of literature**	
Journal article	135
Book chapter	4
Online document	3
Letter	2
PhD thesis	2
Journal article collection	1
**Type of work presented**	
Reflection on a process evaluation	56
Development of a process evaluation approach	38
Systematic review	16
Discussion and recommendations on broad topic of process evaluation	14
Editorial	7
Empirical research	6
Multiple strands of work	3
Literature synthesis	2
Systematic review protocol	2
Handbook	1
Process evaluation guidance	1
Review of reviews	1
**Field of practice**	
Health	143
Education	4
**Country of lead author**	
UK	62
USA	36
Australia	12
Netherlands	10
Denmark	4
South Africa	4
Canada	3
Brazil	1
Finland	1
France	1
Ireland	1
New Zealand	1
Norway	1
Singapore	1
Sweden	1
Zambia	1
Zimbabwe	1
**Focus of literature item**	
Process evaluation approach / framework / guidance	51
Methodological / operational / ethical issues	37
Use of a method / theory in process evaluation	20
Review of process evaluations	19
Value of process evaluation	15
Multiple foci	5
**Type of accompanying evaluation**	
Trial	83
Not specified	43
Standalone process evaluation	9
Pilot/feasibility study	5
Intervention development	2
Pragmatic formative process evaluation	2
Quasi-experimental	2
Health impact assessment	1

### Critical interpretive synthesis overview

**Fig. 2:**
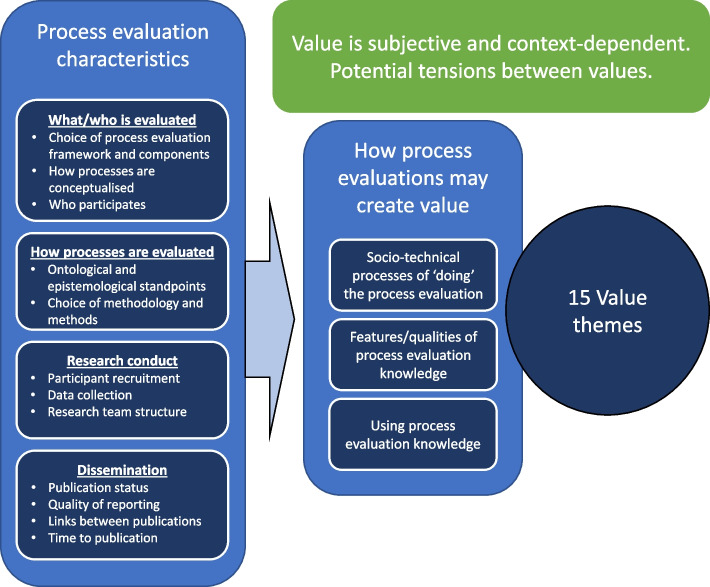
Overview of synthesis findings

Figure 2 provides an overview of the findings of this synthesis.

As shown in Fig. 2, we identified three ways in which process evaluations may create value: 1) through the socio-technical processes of ‘doing’ the process evaluation, 2) through the features/qualities of process evaluation knowledge, and 3) through using process evaluation knowledge.

From these three ways in which process evaluations may create value we identified 15 value themes. Many of these 15 themes included both positive and potentially negative consequences of process evaluations. Value and negative consequences may be created for many different stakeholders, including research participants, researchers, students, funders, research commissioners, intervention staff, organisations, practice settings, research sites, interventions, practice outcomes, and outcome evaluations.

However, as shown in the box describing process evaluation characteristics in Fig. 2, process evaluations may vary widely in terms of 1) which processes are evaluated 2) how these processes are evaluated, 3) the practical conduct of the process evaluation, and 4) how process evaluation knowledge is disseminated. Value is therefore at least partially contingent on the characteristics of individual process evaluations.

Finally, process evaluations are designed, conducted, and their knowledge applied in many different contexts. We found different stakeholders in different contexts may have different perspectives on what is valuable, meaning the value created by process evaluations is subjective. We therefore noted potential tensions and payoffs between certain values.

**Fig. 3:**
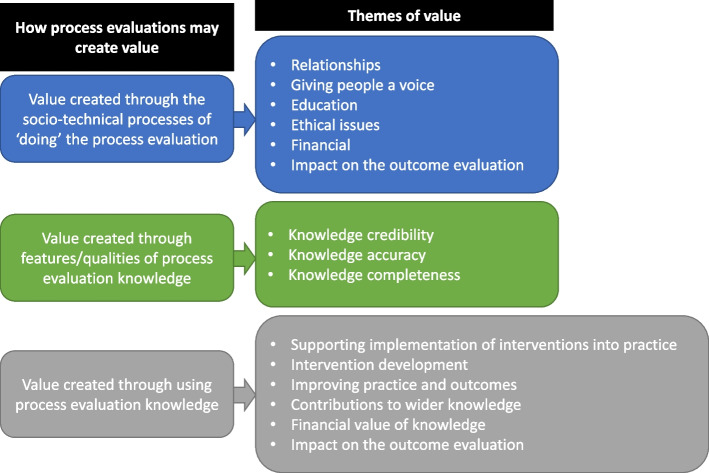
Overview of the themes of value

Figure 3 provides an overview of the themes of value and shows how the themes relate to the three identified ways in which value may be created. We describe these findings in detail in Tables 2, 3, and 4, including subthemes and examples from the synthesised literature. We then end this results section with a discussion of tensions between values.

**Table 2:** Process evaluation value created through the socio-technical processes of ‘doing’ the process evaluation

Theme	Sub-themes	Examples *Denotes potential negative consequences
**Relationships**	**Relationships between process evaluation participants**	Data collection process building trust and identity within a group of process evaluation participants [25] Providing process evaluation participants from different research sites opportunities to network with each other [26] Promoting wider inter-organisational collaboration and learning [27] *Potential negative consequence of status issues and concerns about repercussions between process evaluation participants in group data collection [25]
	**Relationships between researchers and process evaluation participants and other stakeholders**	Activities such as qualitative interviews and stakeholder involvement in research enhancing trust, communication, and a sense of mutual understanding between researchers and process evaluation participants [25, 26, 28] Contribute to broader research/practice partnerships and collaborations [29] *Potential negative consequences of feeding back negative findings to intervention implementers and stakeholders straining relationships and reducing morale and engagement, particularly if not handled sensitively [26, 30] *Possible tension if stakeholders expect feedback during RCTs but this cannot be provided as it would harm the RCT’s ability to establish causality [30, 31] *Potential misunderstandings about purpose of evaluation as grading performance rather than learning opportunities [30]
**Giving people a voice**	**Empowerment or disempowerment of process evaluation participants**	Asking process evaluation participants how to improve interventions signified they were listened to and empowered, however with the important caveat that their views were acted upon [32] Promoting the voices of everybody involved, reflecting dignity and validity of multiple viewpoints [25] Appreciation from process evaluation participants of being asked about their views, experiences, and feelings, about which they were seldom asked [33] Appreciation from process evaluation participants giving opinions in meetings that clinical leaders also present to hear their voice [34] *Potential negative consequence of process evaluation participant disempowerment if views not acted on [32]*, inadequate representation of different stakeholders *[35]*, researcher perspectives privileged *[35]*, researcher use of esoteric language *[36]*, voices perceived as going into a research ‘black hole’ *[34]
**Education**	**Educating students**	Providing students with opportunities to gain experience in research [37] Gaining PhDs through conducting process evaluations [38]
**Ethical issues**	**Consent**	*Conducting observations in settings where some people are not participants in the evaluation [39] *Ethical issues around consent for research use of routinely collected clinical data [40]
	**Confidentiality**	*Confidentiality of individual participant responses, and sensitive handling of information that could be detrimental to others [25, 26]
	**Participant harm**	*Potential emotional ill-effects on process evaluation participants such as embarrassment [1]*, feeling nervous, threatened, uncomfortable being observed *[41] *Disruption and burden to practice settings
	**Acting on process data suggesting problems with the outcome evaluation**	*Ethical dilemmas when process evaluations do not have a formative role but identify problems with an intervention/outcome evaluation [42, 43] Potential role for process evaluations to monitor the ethical conduct of RCTs [42]
**Financial**	**Inefficiency and waste**	*Potential for process evaluations to waste money through inefficiency and collecting too much data [1]
**Impact on the outcome evaluation**	**Increasing likelihood of positive outcome results**	Correcting implementation formatively may increase the likelihood of positive outcome results [11, 39, 44, 45] Realist formative process evaluation in pilot trial resulted in intervention being more adaptable to individual and local contexts and therefore more likely to demonstrate effect in full trial [46] Realist formative process evaluation in pilot trial providing in-depth implementation and delivery knowledge for main trial [46] *Potential for certain process evaluation data collection methods such as in-depth interviews to enhance intervention effects [47] *Potential Hawthorne effects [1]
	**Increasing staff engagement with the evaluation**	Providing feedback to stakeholders through monitoring and quality control may generate enthusiasm, which may be beneficial to the success of the intervention and evaluation [24] Staff delivering interventions are likely to expect and wish to improve their practice [34, 48], and therefore collaboration to formatively improve interventions may have the value of engaging and motivating staff involvement [34] Formative process evaluation may help sustain staff interest and engagement in trials lasting several years [49] Formative improvement of trial processes likely to enhance cooperation of staff collecting process data and timely correction of problems which threaten the evaluation [50]
	**Adding burden to outcome evaluation staff and participants**	*Potential burden of process evaluation data collection on intervention staff and participants [1, 7]
	**Meeting a requirement**	Fulfilling a requirement to include a process evaluation from funding bodies and research commissioners [51, 52], guidance [2, 53], or calls within fields [54, 55]
	**Adding bias to outcome evaluation**	*Outcome evaluators gaining insight into how the intervention is functioning which may bias their interpretation of outcomes [1, 56] *Possibility of unblinded process evaluators accidentally revealing participant allocations to outcome evaluators [1]

**Table 3:** Value related to the features/qualities of process evaluation knowledge

PROCESS EVALUATION VARIABLES	POTENTIAL IMPACTS ON THE VALUE OF PROCESS EVALUATION KNOWLEDGE		
	**Credibility**	**Accuracy**	**Completeness**
**What is evaluated?**	Arguments that process evaluations should be standardised to include set components and enable easier cross-study comparison [1, 5, 24, 57, 58]	Potential for incorrect conclusions to be drawn when insufficient or incorrect processes/participants are included [1, 31] Not taking temporal dimensions into account risks inaccurate interpretation of findings [59] Arguments that process evaluations which conceptualise context, mechanisms of action, and implementation as uni-dimensional, static, and linear may lead to inaccurate conclusions [40, 46, 59–61] Potential for sampled participants/sites to all have had similar experiences so findings do not reflect experiences of whole sample [62]	Arguments for all process evaluations including certain ‘essential’ components [4, 24, 57] Arguments against ‘tick-box’ approach to deciding on components [63] Arguments for stakeholder involvement in selection of processes and participants [1, 44, 64]; potential to miss information through solely basing choices on researcher views [64, 65] Importance of including outcome evaluation processes as well as intervention processes [12, 66–68] Arguments that meaningful interpretation of findings requires analysis of all processes [69, 70] Potential for researchers to only be directed to ‘showcase’ sites [33] Problems using qualitative findings from small numbers of sites to make universal changes to interventions [10] Arguments that process evaluation methods should take account of changes over time, including evolving context [63], intervention teething problems [38, 71], and learning curve effects [55], continuation of intervention beyond trial [4] Debate between using logic models [1] and more complex theoretical models [63, 72–74] to theorise interventions Advocation of using a complex systems perspective to take into account broader systems in which interventions take place [75] Debates about how fidelity should be conceptualised [1, 76, 77] Potential to gain richer understanding through aspects often not investigated, including impact by interaction and emergence [33] and relational dynamics [61]
**How are processes evaluated?**	Doubt from triallists over the credibility of qualitative findings [43], qualitative findings not being properly integrated [78], issues judging whether qualitative or quantitative data are more reliable [79] Difficulties applying nuanced and diverse qualitative findings to interventions developed as uniform in an RCT [10] Potential for rapid qualitative methods to preserve depth of analysis while also providing timely actionable findings [80]	Some qualitative approaches felt to have stronger explanatory capability than others, such as ethnography [34], and the use of theoretical explanatory frameworks [55] Speculative links between factors identified qualitatively and outcomes may not be accurate [68] Potential misleading findings from post-hoc analyses [81, 82] Data collection tools being unable to capture different eventualities of what actually happened [41]	Ability of methods to uncover the unknown [11, 36, 46, 65, 67] Qualitative process evaluations being designed to be subservient to trials [71], avoiding looking for problems [43], framing questions around researchers’ rather than participants’ concerns [83], being undertaken as separate studies [71] Challenges of developing tools to capture all aspects of tailored flexible interventions [41]
**Practical conduct**		Bias introduced during participant recruitment—selective gatekeeping [26], overrepresentation of engaged participants [32, 71, 84] Intervention staff collecting data may introduce bias [1, 40, 48, 71, 82] Routine practice data incomplete or poor quality [12, 40] Low interrater reliability [85], inconsistency between researchers covering different sites [41] Participants may be more willing to honestly express concerns if researchers are separate from the trial [38, 43, 72] Potential for socially desirable narratives [67, 86], recall bias [48, 87], memory limitations [59], inattentive responding [59], and intentional false reporting [59] Analysis of qualitative data with knowledge of outcomes may bias interpretation [13, 88] and result in data dredging [81]	Participants as co-evaluators can strengthen evaluation through gaining richer information [89] Qualitative data analysis without knowledge of outcomes may prevents useful exploration of unexpected outcomes [10, 13] Participants not returning accurate/timely data – in particular lack of motivation in control sites [41]
**Dissemination**		Limited discussion of quality, validity, and credibility in publications [9, 40, 63, 90]	Sometimes not published [1, 78, 91], with no justification of why elements were published over others [71] Process evaluation publications divorced from outcome publications [9, 12, 54, 63, 78, 92]; lengthy time periods between publications [12]

**Table 4:** Value created by using process evaluation knowledge

Theme	Sub-theme	Examples
**Process evaluation knowledge supporting implementation of interventions into practice**	**Improving implementation during the evaluation**	Continuously check and make adjustments to keep interventions ‘on track’ [93] by monitoring and correcting fidelity, adaptations, reach, and/or dose [24, 44, 48, 77, 93, 94]
	**Developing interventions more likely to be implemented successfully**	Formative process evaluation during piloting enhances development of sustainable and adaptable intervention, and develops robust implementation processes increasing likelihood of effectiveness in main trial [46] Formative process evaluation over entire evaluation allows implementation to be optimised and strengthened in real time [60, 89, 95]
	**Informing about transferability to other contexts post-evaluation**	Understanding of the required conditions for interventions to have desired effects, and assessment of intervention transferability to different settings [35, 40, 82, 96] Enable judgement about whether mechanisms would have the same effect in different settings [1, 97] Acceptability of interventions [98] Responses of different subgroups [27]
	**Informing how best to implement the intervention post-evaluation**	Necessary conditions for implementation to be effective in systems, such as new policies [95], allocation of sufficient resources [93] Necessary training and support for intervention deliverers [91, 99–101] How to tailor and adapt interventions in different contexts [40, 53, 62, 81, 83, 99] Strategies and monitoring systems to support implementation [46, 99, 102–104] Informing about relative importance and optimisation of different intervention components [6, 31, 40, 74, 104] Describing how flexible interventions were delivered in evaluation to aid replication [12] Assessment of extent to which intervention is deliverable in practice in the intended way [86]
	**Enhancing likelihood of intervention being implemented in practice post-evaluation**	Engaging stakeholders during process evaluation may contribute to successful implementation by those stakeholders after the evaluation [28, 105] Understanding processes of integrating interventions in dynamic complex settings [106] Providing evidence of feasibility and help convince clinicians and policymakers to adopt controversial but effective interventions [13] Highlighting potential implementation difficulties [13] Providing evidence of how intervention works in different contexts may mean more likely to be adopted in practice [96]
**Process evaluation knowledge informing development of interventions**	**Intervention modification**	Optimisation through revealing reasons for positive outcomes [53, 84] Modification to avoid potentially harmful unintended effects [42, 107] Improvements to acceptability and usability [108, 109] Remove or modify intervention components [70, 91, 99, 110] Inform effective tailoring of interventions to different populations and contexts [62, 84, 99, 111] Improvements to intervention design [86]
	**Developing intervention theory**	Develop, test, and refine intervention theory and causal mechanisms [33, 53, 83, 96, 112]
	**Future intervention design**	Process evaluations providing insights into reasons for ineffective interventions can provide knowledge to inform development of future interventions [90]
**Process evaluation knowledge improving practice and outcomes**	**Improvements during the evaluation**	Formative process evaluations facilitated intervention development and therefore improved practice and outcomes [29, 30, 37, 94, 113] Improving standard care at trial sites by exposing gaps in current provision [12] Designing quality process evaluation from evaluation outset can help examine programme logic and potential for additional positive outcomes [114] Participation in process evaluation may have helped intervention reach goal of empowering youth [32]
	**Improvements after the evaluation**	Process evaluation knowledge ultimately can improve practice and outcomes in groups targeted by interventions through: • Facilitating timely implementation of effective interventions into practice [96, 103, 114] • Providing understanding of how interventions work [115] • Enhancing understanding of complexity [2] Knowledge about patient experience may help clinicians and patients decide which intervention to choose in practice if both are found to have similar effects in an RCT [13] Improving patient centred-care by considering patient views [116] Revealing and addressing inequalities in participant responses which may be masked by aggregate positive trial results [1]
**Process evaluation knowledge contributing to wider knowledge**	**Wider knowledge about interventions**	Inform wider theories about similar interventions [57, 117–120] Generate questions and hypotheses for future research [9] Highlight need for other interventions to target different subgroups [121]
	**Wider knowledge about implementation science**	Knowledge about successful implementation strategies and behaviour change techniques [33, 71, 109, 122, 123] Understanding variation in outcome results according to factors associated with staff delivering interventions may be useful to inform wider research, policy, and practice [55, 81] Contribute insights into what facilitates implementation in public health programs [114]
	**Wider knowledge about contexts**	Contribute to the evidence base about which types of interventions are fruitful to pursue, modify, or should be avoided within certain fields of practice [26, 47]
	**Wider knowledge about research methods**	Methodological and theoretical contributions to process evaluation literature [1, 27, 29, 35, 84, 96, 99, 124, 125] Knowledge about optimal trial designs [90]
**Financial value of process evaluation knowledge**	**Reducing costs of interventions**	Identifying the active ingredients of interventions to inform removing minimally effective components [6, 40, 57] Demonstrating feasibility of implementing intervention in practice without a research grant [93]
	**Justifying cost of evaluations**	By explaining outcome results process evaluations may help justify money spent on trials with outcomes that are not positive [28, 126] Justifying costs of the intervention to funders [127]
	**Informing financial management in wider contexts**	Explaining outcome results may help avoid future expensive mistakes in interventions, theory, and research [67, 92] Understanding the mechanisms of interventions, and how they may affect other areas of health systems, may inform wider health investment [128]
	**Avoiding research waste**	Better provision of information on the influence of context on trial outcomes may help stop trial findings being ignored by policymakers and practitioners [129] The role of process evaluation knowledge in increasing the likelihood of interventions being successfully transferred to practice may be used to justify the expense of process evaluations [67]
	**Ensuring interventions implemented correctly during evaluations**	Formative monitoring and correction of implementation may avoid financial waste through researching interventions which are not implemented correctly [64, 118]
**Value of process evaluation knowledge to the outcome evaluation**	**Adding knowledge not provided by the outcome evaluation**	Unpacking an aggregate positive or negative outcome result which may mask considerable differences in individual benefit of interventions [1, 31, 82] Reasons for variability in outcomes and implementation [95] Qualitative process evaluations may discover unexpected outcomes that are difficult to predict or access using experimental methods [33, 63] Investigating contextual factors not taken into account by outcome evaluation [33, 82] Explaining why interventions do or do not show effect in an outcome evaluation [58, 117] Providing knowledge about how interventions work in practice, including aspects of intervention of which investigators unaware [130], which aspects of intervention most important [109] Providing richer knowledge of how change occurred in ways that mattered to participants [33] Factors contributing to intervention implementation, including negotiations and compromises necessary for successful implementation [34] Unanticipated benefits of interventions [95] *Negative qualitative findings potentially demoralising trial team [92]
	**Increasing the credibility of outcome evaluation methods**	By adding knowledge to address criticisms of limitations of RCTs [81], process evaluations improve the science of RCTs, and help prevent abandonment of RCTs in favour of less rigorous non-experimental or non-randomised research methods [88] Perceptions that process evaluations address tendencies of experimental evaluators to not take into account vital information [1, 38, 54, 55]
	**Improving or interpreting the quality of outcome evaluation results**	Providing summative information about external validity [126, 131] and internal validity [111] Avoiding ‘type III errors’, or ‘false-negative’ trial results, where lack of effect is caused by poor implementation [87, 131] Formative process evaluations may help avoid erroneous trial results through maximising fidelity and therefore internal validity [48, 98, 119] Providing information to enable selection of most appropriate statistical methods for outcome evaluation [5] Providing knowledge about changes in implementation over time [59] and learning curve effects [55] to help interpret outcome results Investigating potentially problematic areas of pragmatic trial design and conduct to support validity of outcome results [12] Through qualitative participatory process evaluation achieving ‘a more robust, rigorous and reliable source of evidence than the single stories that conventional quantitative impact evaluations generate’ [33]
	**Improving outcome evaluation methods**	Formative process evaluation enabling change to outcome study design prior to commencement [95]

### Value created through the socio-technical processes of ‘doing’ the process evaluation

Many social and technical processes are involved in the design, conduct, and dissemination of process evaluation, and thus value and negative consequences may arise from the ‘doing’ of the process evaluation. Examples of socio-technical processes include collecting observational data at a research site, inviting a trial participant to participate in a process evaluation, and designing a questionnaire. These are all carried out by multiple human actors (for example researchers and research participants) using a variety of knowledge products (for example evaluation frameworks and research protocols). In Fig. 2, these processes and actors are summarised under the heading ‘process evaluation characteristics’. Taking a stance that value is situated and formed out of context, the way in which these processes evolve have a direct impact on the value that can be derived from a process evaluation. We identified six themes of value stemming from socio-technical processes:

- Relationships
- Giving people a voice
- Education
- Ethical issues
- Financial
- Impact on the outcome evaluation

Table 2 shows the themes, subthemes, and examples of how socio-technical processes may create value from process evaluations.

### Value related to the features/qualities of process evaluation knowledge

The second way in which process evaluations may create value relates to the features and perceived qualities of the knowledge they produce. The process evaluation characteristics outlined in Fig. 2 clearly lead to different kinds of process evaluation knowledge being produced, for example qualitative or quantitative. We identified three themes of value which relate to the features and qualities of process evaluation knowledge:

- Knowledge credibility
- Knowledge accuracy
- Knowledge completeness

Table 3 outlines how process evaluation variables may impact on the perceived value of the knowledge that is produced.

Inevitably, some of the ways in which process evaluation knowledge may be inaccurate or incomplete described in Table 3 may be unavoidable. For example, it is likely impossible for financial, practical, and ethical reasons for process evaluations to investigate every potentially important aspect of an intervention [1, 41]. Issues such as gatekeeping, self-selection bias, and social desirability bias are research challenges not unique to process evaluations. However, the literature suggests that process evaluation reporting is often suboptimal, with detail on methods lacking, choices about methodology and areas of enquiry not justified [9, 34, 40, 55, 63, 71, 97, 131], and limited discussion of quality, validity, and credibility [9, 40, 63, 90]. This suggests inaccuracy and incompleteness of process evaluation knowledge may not always be acknowledged.

Furthermore, some authors suggest that some process evaluation researchers do not recognise that their methods may be overly simplistic portrayals of reality, and therefore fail to consider important aspects of process [40, 59]. Some papers conceptualised process evaluation components as highly complex, suggesting that methods such as ethnography [34], realist evaluation [46], and the use of theoretical frameworks such as normalisation process theory [132] were necessary to fully capture what was going on. At the opposite end of the spectrum some papers conceptualised process evaluation components simplistically, for example equating whether or not intervention recipients enjoyed intervention components with their effectiveness [91]. A potential negative consequence of process evaluations therefore may be if knowledge is uncritically presented as providing explanations when researchers did not account for all factors or the true level of complexity. For example, assessing single dimensions of implementation may lead to ‘type III errors’ through incorrectly attributing a lack of intervention effect to a single implementation factor, when the actual cause was not investigated [40, 117].

### Value created by using process evaluation knowledge

The third way in which value and negative consequences may be created is through using the knowledge produced by process evaluations. Process evaluation knowledge may be used and applied after the evaluation. It may also be used formatively to make changes to interventions, implementation, contexts, and evaluation processes during the evaluation. Some experimental outcome evaluation methods prevent formative use of knowledge to maintain internal and external validity. We identified six themes of value stemming from the use of process evaluation knowledge:

- Supporting implementation of interventions into practice
- Informing development of interventions
- Improving practice and outcomes
- Contribution to wider knowledge
- Financial value of knowledge
- Impact on the outcome evaluation

These are described along with sub-themes and examples in Table 4.

### Tensions within and between values

As well as identifying how process evaluations may create value and themes of value, we found that the concept of value in process evaluations is subjective and context-dependent, and there are tensions within and between values.

The value of process evaluation is not pre-existing but enacted and created through ongoing negotiation between those with a stake in what is being evaluated. Through designing and conducting a process evaluation and disseminating and using its knowledge, process evaluation actors and knowledge products may directly or indirectly create value and negative consequences for many different stakeholders and bystanders in different contexts. These include people and organisations who participate in research, conduct research, use research findings, receive interventions, work in research and practice settings, fund research, regulate research, or are simply present where process evaluations are being conducted. These groups and organisations have different expectations, values, and needs; and there is also variability within groups and organisations. This creates the potential for tension between expectations, values, and needs of different stakeholders.

We identified two broad perspectives on value. In the first, process evaluations are primarily valued for supporting the scientific endeavour of outcome evaluations, particularly trials. Examples of this include process evaluations being conducted to minimally contaminate or threaten interventions and outcome evaluations, with the generated knowledge applied post-hoc and providing retrospective understanding [87, 118]. Formative monitoring and correction of implementation aims to ensure internal validity [24, 44, 48, 77, 93, 94]. Value is framed around meeting the needs of the outcome evaluation, such as through complementing trial findings [9], and the perceived utility of findings may be contingent on what happens in an outcome evaluation [133]. They are also framed around the needs of researchers and systematic reviewers. For example, calls for them to include set components to make them less daunting to conduct and enable easier cross-study comparison [1, 5, 24, 57, 58].

In the second perspective process evaluations are mostly valued for formatively contributing to intervention development, improving practice, and forging relationships with stakeholders. Evaluating implementation may allow for the adaptation and tailoring of interventions to local contexts [1], which may result in them being more patient-centred [126], with better fit and feasibility in local settings [55]. Process evaluations may be seen as opportunities to utilise methodologies with different ontological and epistemological assumptions to RCTs, with flexible designs that are tailored to the uniqueness of each intervention and setting [34, 67]. These process evaluations are more likely to find multiple nuanced answers, reflecting assumptions that reality is unpredictable and complex, and that interventions are most effective when adapted to different contexts. These seem more concerned with giving participants voices and uncovering messy realities, developing effective sustainable interventions, and through these, improving outcomes [33, 60].

Some authors give examples of process evaluation designs which may capitalise on both perspectives on value. In-depth realist formative process evaluations at the stage of piloting interventions incorporate the benefits of developing and theorising effective, sustainable, adaptable interventions that are tailored to local contexts, which can then be tested in a rigorous outcome evaluation [46]. Pragmatic formative process evaluations theorise interventions which are already in practice and optimise implementation in readiness for outcome evaluations [11, 35].

The literature also contains examples of tensions between these two perspectives. For example, process evaluation methods that enhance engagement with participants may increase the effect of the intervention, which may be seen as desirable [32] or a problematic Hawthorne effect [1]. If data from summative process evaluations reveal problems with interventions or implementation during the evaluation, this can raise ethical and methodological dilemmas about whether to intervene [42, 43]. Riley et al. suggested process data monitoring committees as forums for debating such contentious scenarios to address these issues [43]. Others highlighted the importance of stakeholders having clear expectations about the value that process evaluations may create and when, to avoid tensions stemming from unmet expectation. Examples include establishing clear mandates with intervention staff about when they will receive feedback on their delivery [31] and how their data will improve interventions [89].

## Discussion

### Summary of findings

Process evaluations do not have value a priori. Their value is contingent on the features and qualities of the knowledge they produce, and the socio-technical processes used to produce that knowledge. There is also potential to create consequences that may be perceived negatively. However, there are not simple definitive answers to the questions ‘what kind of value do/should process evaluations create?’ or ‘how do/should process evaluations create value?’. This is because:

- The label ‘process evaluation’ may be applied to many different types of studies producing diverse kinds of knowledge and using diverse socio-technical processes.
- Process evaluations are undertaken in different research and practice contexts in which different kinds of knowledge and socio-technical processes may be perceived as more or less valuable or desirable.
- Process evaluations are undertaken by researchers with differing ontological and epistemological standpoints and research traditions, who have different views on what constitutes high-quality, useful, and valuable knowledge.

### Theoretical considerations

Our analysis shows that part of the challenge of interpreting the value of process evaluation is that researchers and other stakeholders are debating value from different ontological and epistemological starting points. These tensions resonate with the wider literature on qualitative research with quantitative outcome evaluation [13, 45, 134, 135], and how complex interventions should be conceptualised and evaluated [136–138].

There are tensions between values, particularly payoffs between optimising value to outcome evaluations and triallists, and optimising value to intervention development and relationship-building. While the professed aims of both are to improve practice and outcomes for intervention recipients and to advance knowledge, the beliefs about how this is best achieved often differ. For example, process evaluation researchers with a more positivist stance likely believe a positive primary outcome result with high internal validity is most likely to ultimately improve practice and outcomes. They may therefore value process evaluations which minimally contaminate interventions and measure fidelity. Process evaluation researchers with a more interpretivist stance likely believe in-depth understanding of the experiences of intervention recipients is more likely to ultimately improve practice and outcomes. They could therefore value process evaluations which engage participants in more in-depth data collection methods.

While it is beyond the scope of this paper to debate the relative merits of these paradigmatic differences, ontological and epistemological perspectives appear to strongly influence perspectives on what kind of knowledge it is valuable for process evaluations to generate. This demonstrates the importance of making ontological and epistemological perspectives explicit when discussing how to design and conduct process evaluations, for example in process evaluation guidance and frameworks [8].

We also encourage researchers to take stock of these different perspectives on value and critically reflect on whether concentrating value on one perspective potentially misses the opportunities to create value offered by another. For example, through the aim of minimally contaminating interventions are opportunities missed to engage stakeholders who could assist with intervention improvement and post-evaluation implementation? Are there potential ways to combine both approaches to process evaluation? As highlighted in our analysis, in-depth formative process evaluations in the intervention development and feasibility testing stages offer this opportunity [46]. Furthermore, the newly updated Medical Research Council *Framework for evaluating complex interventions* [138] (published after we completed the searches for this review) states *“A trade-off exists between precise unbiased answers to narrow questions and more uncertain answers to broader, more complex questions; researchers should answer the questions that are most useful to decision makers rather than those that can be answered with greater certainty”*. This suggests pragmatic weighing-up of the overall value created by process evaluations will become increasingly significant.

### Practical applications

Our findings have practical applications for researchers designing process evaluations to be intentional in creating value and avoiding negative consequences. We recommend that since process evaluations vary widely, before researchers ask: ‘how do we do this process evaluation? they ask: ‘what do we want to get out of this process evaluation?’. Process evaluations will create value, and potentially negative consequences regardless of whether it is planned, so we suggest purposefully and explicitly preparing to create value in conjunction with stakeholders.

**Fig. 4:**
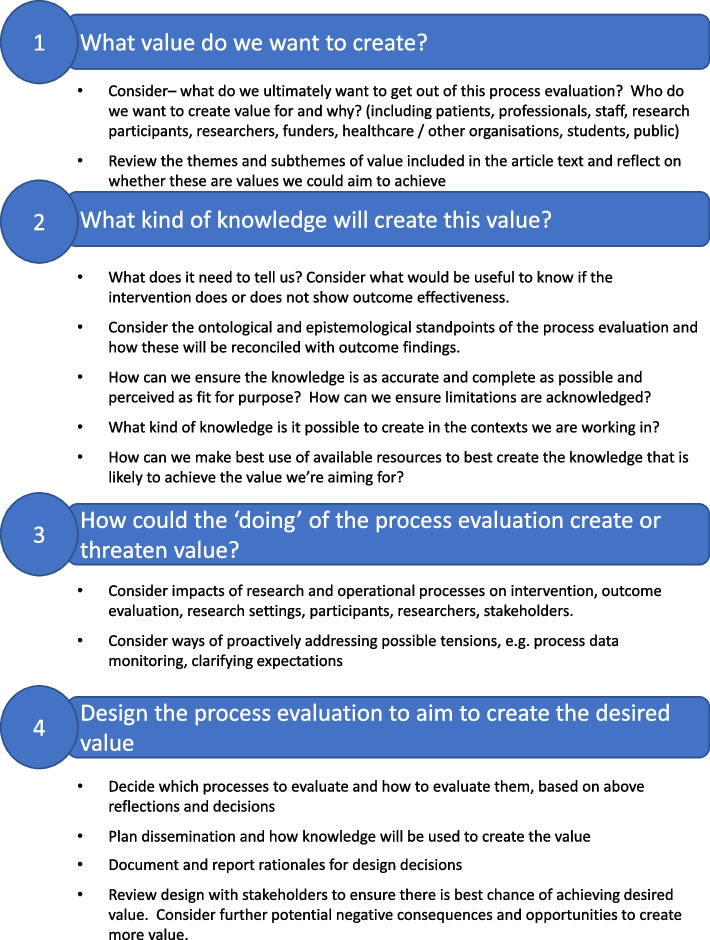
Process evaluation planning framework

Figure 4 shows a planning framework to be used in conjunction with Fig. 3 and the analysis in this paper to aid this process. As would be good practice in any research, we recommend these discussions include as many stakeholders as possible, including intended beneficiaries of research, also reflecting the possible diversity of research backgrounds and epistemological standpoints within research teams. This would help guide decisions around design, conduct, and dissemination by making expectations of value explicit from the outset, addressing potential tensions, and ensure contextual fit. While the nature of any accompanying outcome evaluation will influence expectations of value, it is useful for stakeholders to be aware of potential payoffs and ensure there is a shared vision for creating value. This will likely also aid researchers to narrow the focus of process evaluation to make it more feasible and best allocate resources, as well as highlighting its value to stakeholders without relevant knowledge and experience.

### Strengths and limitations

We included a large number of literature items relating to process evaluations in diverse contexts, which enabled us to synthesise a broad range of perspectives on value and highlight how value may be context dependent. This will enable readers to apply findings to their own contexts. Nonetheless our review does not include all literature that could have been informative, and therefore the values and issues identified are unlikely to be exhaustive. Furthermore, author texts we extracted as data for our review may have been influenced by expectations and limitations of publishing journals. Exploring the concept of value by reviewing the literature only captures perspectives which authors have decided to publish, and other aspects of value are likely to be uncovered through empirical study of process evaluation practice.

Although we have outlined our review methods as explicitly as possible, in line with critical interpretive synthesis the review was by nature interpretive and creative, therefore full transparency about step-by-step methods is not possible [23]. We present our interpretation of this body of literature and acknowledge that this will have been influenced by our pre-existing opinions about process evaluation. Nonetheless our team included researchers from different backgrounds, and through a double-coding process and reflective team discussion ensured we did not unduly focus on one aspect of value or prioritise certain perspectives.

## Conclusions

Process evaluations vary widely and different stakeholders in different contexts may have different expectations and needs. This critical interpretive synthesis has identified potential sources of and themes of value and negative consequences from process evaluations, and critically analysed potential tensions between values. Accommodating all needs and expectations of different stakeholders within a single process evaluation may not be possible, but this paper offers a framework to support an open transparent process to plan and create value and negotiate trade-offs. This supports the developments of joint solutions and, ultimately, generate more value from process evaluations to all.

## Supplementary Information

**Additional file 1.** 

## Data Availability

All data generated or analysed during this study are included in this published article.

## Abbreviations

RCT: randomised controlled trial
