# Supplementary material for: What do we want to get out of this? a critical interpretive synthesis of the value of process evaluations, with a practical planning framework
Source: BMC Med Res Methodol. 2022 Nov 25;22:302. doi: 10.1186/s12874-022-01767-7 (PMC9700891; doi:10.1186/s12874-022-01767-7)
Supplement: Supplementary file 1 — Additional file 1. [file 12874_2022_1767_MOESM1_ESM.docx]

**Additional File 1: Included literature items**

| **Reference** | **Year of publication** | **Country of lead author** | **Type of literature** | **Type of work presented** | **Field of practice** | **Type of accompanying outcome evaluation** | **Focus of literature item** | **Aim (where possible copied and pasted from original article text)** |
| --- | --- | --- | --- | --- | --- | --- | --- | --- |
| Aarestrup et al. (1) | 2014 | Denmark | journal article | development of a PE approach | health | trial | Process evaluation approach / framework / guidance | To present a systematic approach to plan process evaluation of the implementation of randomised multicomponent interventions. |
| Abildgaard et al. (2) | 2016 | Denmark | journal article | reflection on a PE | health | trial | Methodological / operational / ethical issues | To shed light on what type of knowledge of the intervention is gained from qualitative and quantitative process evaluation data. |
| Abraham et al. (3) | 2014 | UK | journal article | discussion and recommendations on broad topic of process evaluation | health | trial | Process evaluation approach / framework / guidance | This article … has developed brief guidance to journal editors to improve the reporting of evaluations of behaviour change interventions, thereby serving as an addition to reporting statements such as CONSORT. |
| Alia et al. (4) | 2015 | USA | journal article | development of a PE approach | health | trial | Process evaluation approach / framework / guidance | To demonstrate how process evaluation is used to assess implementation of, and to provide formative feedback for, a culturally tailored, motivational plus family-based weight loss program. |
| Audrey et al. (5) | 2006 | UK | journal article | reflection on a PE | health | trial | Methodological / operational / ethical issues | We describe the extensive process evaluation embedded within the trial and, rather than focusing on resultant data, we consider the potential for such detailed examination of process to affect the intervention’s delivery, receipt and outcome evaluation. |
| Bakker et al. (6) | 2015 | Netherlands | journal article | development of a PE approach | health | not specified | Process evaluation approach / framework / guidance | Describes the development of a method to concisely summarize the results of process evaluations of complex multi-component interventions. |
| Bakker et al. (7) | 2013 | Netherlands | Letter to journal | discussion and recommendations on broad topic of process evaluation | health | trial | Methodological / operational / ethical issues | n/a |
| Baranowski and Stables (8) | 2000 | USA | journal article | reflection on a PE | health | trial | Process evaluation approach / framework / guidance | The process evaluation results from the nine 5-a-Day projects were overviewed. |
| Biron and Karanika-Murray (9) | 2014 | Canada | journal article | discussion and recommendations on broad topic of process evaluation | health | not specified | Process evaluation approach / framework / guidance | We discuss how intervention process evaluation (IPE), an emerging field in intervention research, can enhance our understanding of why organizational interventions for stress succeed or fail. |
| Boeije et al. (10) | 2015 | Netherlands | journal article | discussion and recommendations on broad topic of process evaluation | health | trial | Methodological / operational / ethical issues | This paper addresses the methodological challenges that accompany the use of a combination of research methods to evaluate complex interventions. |
| Brand et al. (11) | 2019 | UK | journal article | reflection on a PE | health | pilot/feasibility study | Methodological / operational / ethical issues | We describe and illustrate how a realist approach to formative process evaluation develops contextualised intervention theory that can underpin more adaptable and scalable interventions. We discuss challenges and benefits of this approach. |
| Branscum and Hayes (12) | 2013 | USA | journal article | review of reviews | health | not specified | Review | Reports the use of process evaluations in childhood obesity prevention interventions implemented over the past three decades. |
| Britton et al. (13) | 1998 | UK | Letter to journal | discussion and recommendations on broad topic of process evaluation | health | trial | Value of process evaluation | n/a |
| Buckley and Sheehan (14) | 2009 | Australia | journal article | reflection on a PE | health | not specified | Process evaluation approach / framework / guidance | Sought to provide examples of how to operationalize a process evaluation of an effective programme. |
| Bunce et al. (15) | 2014 | USA | journal article | reflection on a PE | health | trial | Methodological / operational / ethical issues | The specific methods used in such ethnographic process evaluations are rarely presented in detail; our objective is to stimulate a conversation around the successes and challenges of specific data collection methods in health care settings. |
| Butterfoss (16) | 2006 | USA | journal article | literature synthesis | health | not specified | Methodological / operational / ethical issues | This review provides a synthesis of published public health and social science literature to determine how process evaluation has been used to examine community participation and its intermediary role in health and social change outcomes. |
| Byng et al. (17) | 2005 | UK | journal article | reflection on a PE | health | trial | Methodological / operational / ethical issues | This article explores how a relatively low-cost evaluation, using qualitative methods and Pawson and Tilley’s realistic evaluation (RE) framework (1997) can both help explain the results of the trial and provide generalizable conclusions about service development of relevance to practitioners and policy makers. |
| Byng et al. (18) | 2008 | UK | journal article | reflection on a PE | health | trial | Value of process evaluation | This paper builds a picture of how the intervention, as a whole, had its effects and how the process evaluation adds meaning to the results of the trial. |
| Chandler et al. (19) | 2015 | UK | journal article | empirical research | health | trial | Use of a method / theory in process evaluation | To examine the application of core concepts from Complexity Theory to explain the findings from a process evaluation undertaken in a trial evaluating implementation strategies for recommendations about reducing surgical fasting times. |
| Cheng and Metcalfe (20) | 2018 | Singapore | journal article | editorial | health | trial | Methodological / operational / ethical issues | n/a |
| Chrisman et al. (21) | 2002 | USA | journal article | reflection on a PE | health | standalone PE | Process evaluation approach / framework / guidance | This article reports the design we constructed and how it has worked so far. |
| Cornwall and Aghajanian (22) | 2017 | UK | journal article | reflection on a PE | health | standalone PE | Use of a method / theory in process evaluation | This article considers the contribution participatory process evaluation can make to impact assessment, using a case study of a study carried out to evaluate. |
| Crutzen et al. (23) | 2012 | Netherlands | journal article | reflection on a PE | health | not specified | Use of a method / theory in process evaluation | This study aimed to demonstrate the potential of Google Analytics as a process evaluation method for Internet delivered interventions, using a website about sexual health as an example. |
| Cunningham et al. (24) | 2000 | USA | journal article | reflection on a PE | health | quasi-experimental | Methodological / operational / ethical issues | Included in the paper are the purposes of each process method, problems identified, and their resolution. Suggestions are made for use of process evaluation in community health education programs. |
| De Silva et al. (25) | 2014 | UK | journal article | development of a PE approach | health | not specified | Process evaluation approach / framework / guidance | We propose a theory-driven approach to the design and evaluation of complex interventions by adapting and integrating a programmatic design and evaluation tool, Theory of Change (ToC), into the MRC framework for complex interventions. |
| Diaz et al. (26) | 2014 | USA | journal article | development of a PE approach | health | not specified | Process evaluation approach / framework / guidance | To use a newly devised set of criteria to review the study design and scope of collection of process, outcomes and contextual data for evaluations and implementation research of integrated community case management (iCCM) in Sub–Saharan African. |
| Ekambareshwar et al. (27) | 2021 | Australia | journal article | systematic review | health | not specified | Review | We aimed to summarise the literature in early childhood obesity prevention interventions delivered via telephone or text messages for evidence of application of process evaluation primarily to evaluate stakeholders’ acceptability of interventions. |
| Ellard and Parsons (28) | 2010 | UK | book chapter | discussion and recommendations on broad topic of process evaluation | health | not specified | Methodological / operational / ethical issues | This chapter discusses issues relating to formative process evaluation and process evaluation; explores the research methods used; and discusses the integration of process and outcome data. |
| Ellard et al. (29) | 2011 | UK | journal article | reflection on a PE | health | trial | Methodological / operational / ethical issues | A process evaluation was commissioned alongside the trial and we report the protocol for this process evaluation. |
| Evans et al. (30) | 2015 | UK | Journal article | editorial | health | pragmatic formative process evaluation | Process evaluation approach / framework / guidance | n/a |
| Ferm et al. (31) | 2018 | Denmark | journal article | development of a PE approach | health | trial | Process evaluation approach / framework / guidance | The aims of this paper were, first, to develop a model for a comprehensive quantitative implementation evaluation and, second, to operationalize it in the process evaluation of the stepped-wedge cluster randomized controlled trial. |
| Francis et al. (32) | 2008 | UK | journal article | reflection on a PE | health | trial | Use of a method / theory in process evaluation | This study illustrates the use of theory-based process evaluation to uncover processes underlying change in implementation trials. |
| Franzen et al. (33) | 2009 | USA | journal article | reflection on a PE | health | standalone PE | Value of process evaluation | The present study illustrates how a systematic process evaluation study can improve program activities. |
| French et al. (34) | 2020 | UK | journal article | systematic review | health | trial | Review | We aimed to describe the frequency, characteristics, labelling, value, practical conduct issues, and accessibility of published process evaluations within pragmatic RCTs in health services research. |
| Frost et al. (35) | 2020 | UK | journal article | reflection on a PE | health | trial | Use of a method / theory in process evaluation | We used Social Practice Theory to guide a Type 1 Hybrid Trial: a mixed methods process evaluation of a complex intervention for heart failure. The objective of this paper is to explore the value of Social Practice Theory for implementation science. |
| Gale et al. (36) | 2019 | USA | journal article | reflection on a PE | health | standalone PE | Use of a method / theory in process evaluation | We present our method for conducting a framework-guided rapid analysis (RA) and a comparison of these findings to an in-depth analysis of interview transcripts. |
| Gensby et al. (37) | 2018 | Norway | journal article | development of a PE approach | health | trial | Process evaluation approach / framework / guidance | This study design article is a proposal on a theory-driven and interactive research methodology for a process evaluation of a pragmatic intervention trial, known as STAiR. |
| Gobat et al. (38) | 2021 | UK | journal article | reflection on a PE | health | pragmatic formative process evaluation | Process evaluation approach / framework / guidance | This study reports a novel approach to modelling and refining the programme theory of a whole-school restorative approach, alongside plans to scale up through a national educational infrastructure in order to support robust scientific evaluation. |
| Grant et al. (39) | 2012 | UK | journal article | reflection on a PE | health | trial | Methodological / operational / ethical issues | This paper presents the mixed-method process evaluation protocol of a cluster randomized trial, drawing on a framework designed by the authors. |
| Grant et al. (40) | 2013 | UK | journal article | development of a PE approach | health | trial | Process evaluation approach / framework / guidance | Develop our own framework for designing process evaluations of cluster-randomised controlled trials. |
| Grant et al. (41) | 2016 | UK | Journal article | editorial | health | trial | Use of a method / theory in process evaluation | n/a |
| Grant et al. (42) | 2020 | UK | journal article | reflection on a PE | health | trial | Use of a method / theory in process evaluation | We address this gap in the literature by presenting a number of important considerations for process evaluation using a case study design. |
| Gray and Shaw (43) | 2019 | Canada | journal article | reflection on a PE | health | not specified | Methodological / operational / ethical issues | The purpose of this paper is to explore summative, process and developmental approaches to evaluating complex interventions to determine how to best test this mess. |
| Griffin et al. (44) | 2014 | UK | journal article | development of a PE approach | health | trial | Process evaluation approach / framework / guidance | In this paper, we add to the current literature by describing a comprehensive approach to process evaluation undertaken in a trial of a complex, primary school-based obesity prevention intervention; |
| Griffin et al. (45) | 2017 | UK | journal article | development of a PE approach | health | trial | Process evaluation approach / framework / guidance | The aim of this paper is twofold: 1) to demonstrate a replicable method of process evaluation data synthesis for use by other complex health intervention researchers, and 2) to present the results of the WAVES study process evaluation, demonstrating how the intervention was delivered and received. |
| Harachi and Fleming (46) | 1999 | USA | journal article | reflection on a PE | health | not specified | Use of a method / theory in process evaluation | This article illustrates the utilization of process measures from a multicomponent school-based prevention program to examine implementation of a teaching staff development intervention, and the program's underlying theoretical basis. |
| Harvey and Jan (47) | 2017 | Australia | journal article | editorial | health | trial | Multiple | n/a |
| Hatcher and Bonell (48) | 2016 | South Africa | journal article | editorial | health | trial | Value of process evaluation | n/a |
| Havermans et al. (49) | 2016 | Netherlands | journal article | systematic review | health | not specified | Review | This systematic review aimed to explore which process variables are used in stress management intervention (SMI) evaluation research. |
| Hawe et al. (50) | 2004 | Canada | journal article | reflection on a PE | health | trial | Methodological / operational / ethical issues | In this paper, we describe a combination of qualitative and quantitative methods in place to track the unfolding of a large scale primary care and community development intervention in maternal health in Australia. |
| Haynes et al. (51) | 2014 | Australia | journal article | reflection on a PE | health | trial | Use of a method / theory in process evaluation | This protocol provides a worked example of how to embed process evaluation in the design and evaluation of a complex intervention trial. |
| Haynes et al. (52) | 2016 | Australia | journal article | development of a PE approach | health | trial | Process evaluation approach / framework / guidance | In this paper, we identify and respond to the fidelity assessment challenges posed by novel contextualised interventions (i.e. interventions that are informed by composite social and psychological theories and which incorporate standardised and flexible components in order to maximise effectiveness in complex settings). |
| Helitzer et al. (53) | 2000 | USA | journal article | reflection on a PE | health | not specified | Methodological / operational / ethical issues | In examining this component of the process evaluation, the paper presents the important benefits of monitoring implementation and providing early feedback to the training process. |
| Hickey et al. (54) | 2016 | Ireland | journal article | reflection on a PE | health | trial | Methodological / operational / ethical issues | In this paper, a process evaluation protocol for an early years parenting intervention, the Parent and Infant (PIN) program, is described. |
| Hulscher et al. (55) | 2003 | Netherlands | journal article | development of a PE approach | health | not specified | Multiple | This paper explores the purpose and value of process evaluation on QI interventions and addresses the issue of what data should be collected (“what to measure”) and data collection methods (“how to measure”). |
| Humphrey et al. (56) | 2016 | UK | online document | handbook | education | not specified | Process evaluation approach / framework / guidance | The purpose of this introductory handbook is to provide guidance to Endowment Foundation (EEF) evaluators (and, indeed, other researchers) on how to conduct high-quality implementation and process evaluations (IPE) at the various stages of intervention development and testing (e.g. pilot, efficacy, effectiveness). |
| Humphrey et al. (57) | 2016 | UK | online document | literature synthesis | education | not specified | Review | To draw together existing knowledge regarding the aims, functions and methods of implementation and process evaluation (IPE) in relation to educational interventions, with a view to subsequently informing guidelines for researchers. |
| Irvine et al. (58) | 2012 | UK | journal article | reflection on a PE | health | pilot/feasibility study | Process evaluation approach / framework / guidance | This study assesses the utility of novel techniques for process evaluation involving no face to face contact. |
| Jansen et al. (59) | 2009 | Netherlands | journal article | systematic review | health | trial | Review | This article aims to review the contribution of qualitative research to developing community based interventions in primary care evaluated by means of the pragmatic trial methodology. |
| Kelley et al. (60) | 2001 | USA | journal article | reflection on a PE | health | standalone PE | Value of process evaluation | To use process evaluation methods to describe the development of a hospital-based mental health clinic for children facing medical stressors. |
| Kostamo et al. (61) | 2019 | Finland | journal article | reflection on a PE | health | trial | Use of a method / theory in process evaluation | To describe how to use the critical incident technique (CIT) to conduct in qualitative process evaluation to identify events, including intervention elements, which LMI trial participants perceived to enable or support behavior change. |
| Koutsouris et al. (62) | 2019 | UK | journal article | reflection on a PE | education | trial | Value of process evaluation | The significance of a process evaluation for understanding randomised controlled trials (RCTs) of complex teaching interventions in a classroom setting is discussed in relation to the evaluation of the Integrated Group Reading (IGR) programme trial |
| Lee et al. (63) | 2011 | Canada | journal article | reflection on a PE | health | pilot/feasibility study | Process evaluation approach / framework / guidance | This article describes a fresh, ethnographically informed approach focused on alignments of project components in a pilot multisite, multilevel community-based falls prevention study in Canada |
| Lee et al. (64) | 2013 | USA | journal article | development of a PE approach | health | not specified | Process evaluation approach / framework / guidance | To use and review a conceptual model of process evaluation and to examine the implementation of a nutrition education curriculum, |
| Leeming et al. (65) | 2016 | UK | journal article | discussion and recommendations on broad topic of process evaluation | health | trial | Process evaluation approach / framework / guidance | In this paper, we discuss the limitations of evidence gained from measuring large‐scale outcomes in RCTs and argue that greater use of qualitative research can enhance understanding of key processes in supporting breastfeeding. |
| Legrand et al. (66) | 2018 | France | journal article | empirical research | health | not specified | Methodological / operational / ethical issues | The aim of this study was to describe the practices of health promotion professionals when evaluating interventions and their transferability and to identify these professionals’ needs in relation to a tool that will guide them during monitoring and evaluation. |
| Leontjevas et al. (67) | 2012 | Netherlands | journal article | development of a PE approach | health | trial | Methodological / operational / ethical issues | In this article, a model is presented that divides process evaluation data into first and second order process data. |
| Limbani et al. (68) | 2019 | South Africa | journal article | reflection on a PE | health | trial | Methodological / operational / ethical issues | We report on challenges and lessons learned from undertaking process evaluation of seven hypertension intervention trials funded through the Global Alliance of Chronic Diseases (GACD). |
| Linnan and Steckler (69) | 2002 | USA | book chapter | development of a PE approach | health | not specified | Use of a method / theory in process evaluation | n/a |
| Liu et al. (70) | 2016 | Australia | journal article | systematic review protocol | health | trial | Review | We aim to consolidate the methodology and methods from process evaluations of complex interventions in PHC and their findings of facilitators and barriers to intervention implementation. |
| Liu et al. (71) | 2019 | Australia | journal article | systematic review | health | trial | Review | We synthesised the methods used in PEs of primary care interventions, and their main findings on implementation barriers and facilitators. |
| Lorencatto et al. (72) | 2016 | UK | journal article | development of a PE approach | health | trial | Process evaluation approach / framework / guidance | This protocol presents methods for assessing fidelity across five dimensions proposed by the Behaviour Change Consortium fidelity framework, including intervention designer-, provider- and recipient-levels. |
| Lytle et al. (73) | 1994 | USA | journal article | reflection on a PE | health | trial | Methodological / operational / ethical issues | This paper discusses the challenges faced when collecting process evaluation information in a school-based, multicenter field trial. |
| Maar et al. (74) | 2017 | Canada | journal article | development of a PE approach | health | trial | Process evaluation approach / framework / guidance | We present a framework for the process evaluations for mHealth interventions in multiple cultural settings. |
| Manchaiah et al. (75) | 2014 | UK | journal article | discussion and recommendations on broad topic of process evaluation | health | not specified | Methodological / operational / ethical issues | The main focus of this paper is to discuss the importance of “evaluating the process of change” (i.e., process evaluation) in people with disability by studying their lived experiences. |
| Masterson-Algar (76) | 2016 | UK | PhD thesis | multiple strands of work | health | trial | Multiple | The aim of this study is to advance thinking and practice in process evaluation and clinical trial methodology within the field of neurological rehabilitation. |
| Masterson-Algar et al. (77) | 2016 | UK | journal article | systematic review | health | trial | Review | To systematically review how process evaluations are currently designed, what methodologies are used and how are they developed alongside or within neurological rehabilitation trials. |
| Masterson-Algar et al. (78) | 2018 | UK | journal article | empirical research | health | trial | Process evaluation approach / framework / guidance / guidance | This paper reports on the development of new consensus guidelines that address the specific challenges of conducting process evaluations alongside clinical trials of rehabilitation interventions. |
| May et al. (79) | 2007 | UK | journal article | development of a PE approach | health | trial | Process evaluation approach / framework / guidance | The paper develops this by first discussing the development of the theoretical model, and then applies it to two case studies of complex trials that combine both treatment and organizational interventions in primary care. In the conclusion, the implications of such models for the development of process evaluations are discussed. |
| May et al. (80) | 2018 | UK | journal article | systematic review | health | not specified | Review | The aims of this review were to review this literature; to identify and characterise the uses and limits of NPT in research on the implementation and integration of healthcare interventions; and to explore NPT’s contribution to understanding the dynamics of these processes |
| Mbuya et al. (81) | 2015 | Zimbabwe | journal article | development of a PE approach | health | trial | Process evaluation approach / framework / guidance | In this article, we present the SHINE PIP including definitions and measurements of key mediating domains, and discuss the implications of this approach for randomized trials. |
| McGill et al. (82) | 2020 | UK | journal article | systematic review | health | not specified | Process evaluation approach / framework / guidance | This systematic review aims to analyze published examples of process evaluations that utilize qualitative methods that involve a complex systems perspective and proposes a framework for qualitative complex system process evaluations. |
| McGraw et al. (83) | 1994 | USA | journal article | development of a PE approach | health | trial | Use of a method / theory in process evaluation | The process evaluation system used in the Child and Adolescent Trial for Cardiovascular Health (CATCH) is presented in this paper. |
| McIntyre et al. (84) | 2020 | UK | journal article | systematic review | health | trial | Review | In this study context, we reviewed (a) the proportion of process evaluations citing theoretical approaches, (b) which theoretical approaches were cited, and (c) whether and how theories were used. |
| Moore (85) | 2010 | UK | PhD thesis | multiple strands of work | health | trial | Process evaluation approach / framework / guidance | This thesis develops a mixed-method framework exploring programme theory, diffusion, implementation, participant experiences and reach, which is applied to the evaluation of the Welsh National Exercise Referral Scheme (NERS). |
| Moore et al. (86) | 2014 | UK | online document | process evaluation guidance | health | not specified | Process evaluation approach / framework / guidance | This document provides researchers, practitioners, funders, journal editors and policy-makers with guidance in planning, designing, conducting and appraising process evaluations of complex interventions. |
| Moore et al. (87) | 2014 | UK | Journal article | editorial | health | not specified | Multiple | n/a |
| Moore et al. (88) | 2015 | UK | journal article | development of a PE approach | health | not specified | Process evaluation approach / framework / guidance | In this article, we provide an overview of the new framework and summarise our practical recommendations using one of the case studies as an example. |
| Morgan-Trimmer (89) | 2015 | UK | journal article | discussion and recommendations on broad topic of process evaluation | health | not specified | Methodological / operational / ethical issues | This article reflects on the current state of process evaluations of health behavior interventions and argues that evaluation practice in this area could be improved by drawing on the social science literature to a greater degree. |
| Morgan-Trimmer and Wood (90) | 2016 | UK | journal article | discussion and recommendations on broad topic of process evaluation | health | not specified | Use of a method / theory in process evaluation | This article outlines the contribution that ethnography could make to process evaluations for trials of complex health behaviour interventions. |
| Morrels-Samuels et al. (91) | 2018 | USA | journal article | development of a PE approach | health | trial | Process evaluation approach / framework / guidance | The purpose of this article is to present methods used to document and assess the implementation of the Youth Empowerment Solutions (YES) program. |
| Munodawafa et al. (92) | 2018 | South Africa | journal article | systematic review | health | not specified | Review | This review seeks to answer two main questions: (i) to what extent are qualitative process evaluations conducted on task shared interventions for perinatal depression in LAMICs; and (ii) what is the best way to synthesize emergent themes from the process evaluations with the MRC framework for conducting process evaluations [20]? |
| Munro and Bloor (93) | 2010 | UK | journal article | discussion and recommendations on broad topic of process evaluation | health | trial | Value of process evaluation | not stated |
| Murdoch (94) | 2016 | UK | journal article | development of a PE approach | health | trial | Process evaluation approach / framework / guidance | In this paper I propose an alternative approach to the design, implementation and analysis of process evaluations for complex health interventions through a consideration of trial protocols as textual documents, distributed and enacted at multiple contextual levels. |
| Murta et al. (95) | 2007 | Brazil | journal article | systematic review | health | not specified | Review | To conduct a systematic review of workplace stress management intervention studies that have incorporated process evaluation. |
| Murtagh et al. (96) | 2007 | UK | journal article | reflection on a PE | health | trial | Methodological / operational / ethical issues | To understand participants’ experiences and understandings of the interventions in the trial of a computerised decision support tool in patients with atrial fibrillation being considered for anti-coagulation treatment. |
| Nagy et al. (97) | 2007 | USA | journal article | reflection on a PE | health | standalone PE | Value of process evaluation | In this manuscript we report on the development, implementation, results, and lessons learned from a process evaluation plan initiated during the planning period for the Alabama Racial and Ethnic Approaches to Community Health (REACH 2010) program. |
| Nielsen and Abildgaard (98) | 2013 | UK | journal article | development of a PE approach | health | not specified | Process evaluation approach / framework / guidance | In this paper, we present an evaluation framework based on recent intervention research and process-oriented organization theory. |
| Nielsen and Randall (99) | 2013 | Denmark | journal article | development of a PE approach | health | not specified | Process evaluation approach / framework / guidance | By drawing on existing intervention literature we present an evidence-based model containing three levels of elements that appear to be crucial in process evaluation. |
| Nielsen et al. (100) | 2018 | USA | journal article | reflection on a PE | health | trial | Value of process evaluation | Here, we report on some of the key findings from the PE conducted 1 year after programme implementation began (Olney, Behrman, Iruhiriye, van den Bold, & Pedehombga, 2013), how the findings were used to refine programme implementation and rectify weaknesses in real time through a series of consultations |
| Oakley et al. (101) | 2004 | UK | journal article | reflection on a PE | health | trial | Methodological / operational / ethical issues | The article describes the methods used to collect process data, and some of the challenges involved. |
| Oakley et al. (102) | 2006 | UK | journal article | development of a PE approach | health | trial | Process evaluation approach / framework / guidance | This paper outlines a framework for using process evaluation as an integral element of RCTs. |
| O'Cathain et al. (103) | 2014 | UK | journal article | multiple strands of work | health | trial | Value of process evaluation | To systematically explore how qualitative research is being used with trials and identify ways of maximising its value to the trial aim of providing evidence of effectiveness of health interventions. |
| Odendaal et al. (104) | 2008 | South Africa | journal article | reflection on a PE | health | trial | Value of process evaluation | This article reviews the methods in a process evaluation of a home visitation programme. |
| Ottoson et al. (105) | 2000 | USA | journal article | reflection on a PE | health | not specified | Value of process evaluation | The purpose of this study was to explore process or contextual aspects of the work setting and their relationship to learning outcomes following CPE. |
| Palmer et al. (106) | 2016 | Australia | journal article | development of a PE approach | health | trial | Methodological / operational / ethical issues | This paper provides a description of a nested process evaluation design using mixed-methods to inform a cluster randomized controlled trial. It builds on current debates about the need to better systematize process evaluation data collection, analysis and reporting. |
| Parker et al. (107) | 2019 | USA | Journal article | editorial | health | pilot/feasibility study | Value of process evaluation | n/a |
| Parrott and Carman (108) | 2019 | USA | journal article | reflection on a PE | health | not specified | Methodological / operational / ethical issues | In this research and practice note, we reflect on our evaluation experiences with a public - private partnership designed to scale up a health and wellness program within a large, urban school district at ten elementary schools. |
| Platt et al. (109) | 2004 | UK | book chapter | discussion and recommendations on broad topic of process evaluation | health | not specified | Methodological / operational / ethical issues | This chapter introduces the purposes, focus, and methods of process evaluation and explores some issues in the application of process evaluation using examples from two research projects. |
| Raine et al. (110) | 2016 | UK | journal article collection | discussion and recommendations on broad topic of process evaluation | health | not specified | Multiple | This collection of essays is intended to address at least the first part of the paradox, the relative lack of consensus about how to evaluate changes in services. |
| Ramsay et al. (111) | 2010 | UK | journal article | reflection on a PE | health | trial | Use of a method / theory in process evaluation | To illustrate the applicability of causal methods within randomised trials, we undertook a theory-based process evaluation study within an implementation trial to explore whether the cognitions of primary care doctors’ predicted their test requesting behaviours and, secondly, whether the trial results were mediated by the theoretical constructs. |
| Redmond et al. (112) | 2017 | USA | journal article | development of a PE approach | health | trial | Process evaluation approach / framework / guidance | The aim of this study was to describe a refined process evaluation model adapted for Web-based settings and used to assess the implementation of a Web-based intervention aimed to increase organ donation among African Americans. |
| Renger and Foltysova (113) | 2013 | USA | journal article | reflection on a PE | health | standalone PE | Process evaluation approach / framework / guidance | The purpose of this study is to report on our efforts at assessing the feasibility of capturing program planning phase deliberations and using them to design a process evaluation capable of providing information for making decisions about both quality control and quality improvement. |
| Reynolds et al. (114) | 2014 | UK | journal article | empirical research | health | trial | Process evaluation approach / framework / guidance | In this paper, we will draw on our experiences of ‘doing’ evaluation in a research context to present lessons learned for negotiating the reality of evaluation and reflecting on the subsequent implications for interpreting trial outcomes. |
| Ribeiro et al. (115) | 2019 | New Zealand | journal article | systematic review | health | trial | Review | This systematic review is designed to answer the following research question: How are process evaluation of complex interventions tested in RCTs in musculoskeletal disorders being conducted? |
| Riley et al. (116) | 2005 | Australia | journal article | reflection on a PE | health | trial | Methodological / operational / ethical issues | This paper presents issues which arose in the conduct of qualitative evaluation research within a cluster randomized, community-level, preventive intervention trial. |
| Roberts-Gray et al. (117) | 2017 | USA | journal article | development of a PE approach | health | trial | Process evaluation approach / framework / guidance | To examine the utility of structuring the trial’s process evaluation to forecast use, sustainability, and readiness of the intervention for wider dissemination and implementation. |
| Roe and Roe (118) | 2004 | USA | journal article | development of a PE approach | health | not specified | Process evaluation approach / framework / guidance | This article describes dialogue boxes, a process evaluation tool that has proven extremely useful in diverse health promotion program and planning efforts. |
| Rycroft-Malone et al. (119) | 2018 | UK | journal article | reflection on a PE | health | trial | Use of a method / theory in process evaluation | This paper presents an exemplar of a multi-country realist process evaluation that was embedded in the first international randomised controlled trial evaluating two types of facilitation for implementing urinary continence care recommendations. |
| Saarijärvi et al. (120) | 2020 | Sweden | journal article | reflection on a PE | health | trial | Methodological / operational / ethical issues | In this article, process evaluation is described according to the Medical Research Council guidance and its use exemplified through a randomized controlled trial evaluating the effectiveness of a transition program for adolescents with chronic conditions. |
| Saunders et al. (121) | 2005 | USA | journal article | development of a PE approach | health | not specified | Process evaluation approach / framework / guidance | The purpose of this article is to describe and illustrate the steps involved in developing a process evaluation plan for any health promotion program. |
| Scantlebury et al. (122) | 2020 | UK | journal article | reflection on a PE | health | trial | Process evaluation approach / framework / guidance | The paper aims, through a qualitative process evaluation, to explore some of the factors that may have affected the delivery of the REFORM intervention and highlight how project-specific fidelity can be assessed using a truly mixed-methods approach when informed by qualitative insights. |
| Scott et al. (123) | 2014 | Canada | journal article | systematic review protocol | health | not specified | Review | This study focuses on improving process evaluations by synthesizing current evidence on process evaluations conducted alongside experimental designs for evaluating KT interventions. |
| Scott et al. (124) | 2019 | Canada | journal article | systematic review | health | not specified | Review | This study synthesizes current evidence of KT process evaluations to provide future methodological recommendations. |
| Segrott et al. (125) | 2017 | UK | journal article | reflection on a PE | health | trial | Use of a method / theory in process evaluation | In doing so, this paper aids interpretation of outcome effects within the trial, develops our understanding of how SFP 10–14 could be implemented in a UK setting, and assesses the potential for ENPT to contribute to understanding of implementing social interventions within complex systems. |
| Sharma et al. (126) | 2017 | Canada | journal article | development of a PE approach | health | trial | Process evaluation approach / framework / guidance | Building from previous frameworks, we illustrate a methodology to evaluate implementation processes of the complex CLIP intervention, assess mechanisms of impact and identify emerging unintended causal pathways. |
| Shepherd et al. (127) | 2019 | Australia | journal article | development of a PE approach | health | trial | Process evaluation approach / framework / guidance | This paper describes methods of defining and measuring outcomes of implementation success. |
| Siddiqui et al. (128) | 2018 | UK | journal article | reflection on a PE | education | trial | Use of a method / theory in process evaluation | In this paper, we explain the concept of aggregated trials in schools, and the protocols we followed for the process evaluation in order to gain more information than usual on the feasibility of conducting aggregated trials. |
| Simuyemba et al. (129) | 2020 | Zambia | journal article | reflection on a PE | health | standalone PE | Methodological / operational / ethical issues | This article highlights complexities of this type of real-time evaluation and shares lessons learnt on conducting such evaluation from the Zambian experience. |
| Stappers et al. (130) | 2020 | Netherlands | journal article | reflection on a PE | health | health impact assessment | Methodological / operational / ethical issues | The aim of this paper is to explore and describe the role of context in explaining the developments within the integrated urban reconstruction project and its presumed health effects over time, and to draw implications for evaluation theory and methodology such as spatial or urban health impact assessment. |
| Strange et al. (131) | 2006 | UK | journal article | reflection on a PE | health | trial | Methodological / operational / ethical issues | This article has three aims: to outline the methods developed to link process and outcome data in the RIPPLE trial; to present the findings of this analysis; and to explore some of the methodological issues that arose, especially in using the process data to explain the impact on trial outcomes. |
| Tolma et al. (132) | 2009 | USA | journal article | reflection on a PE | health | intervention development | Methodological / operational / ethical issues | In this article, we describe the process evaluation planning that took place during the development of an action plan by a newly developed Turning Point community partnership. |
| Tolma et al. (133) | 2011 | USA | journal article | reflection on a PE | health | intervention development | Process evaluation approach / framework / guidance | To describe the systematic approach to process evaluation of a Turning Point initiative in central Oklahoma during the formation stage. |
| Tonkin-Crine et al. (134) | 2016 | UK | journal article | development of a PE approach | health | trial | Use of a method / theory in process evaluation | The current study aimed to follow a triangulation protocol to integrate mixed methods data previously collected in order to see whether such an approach could further inform the findings of the original process evaluation of the trial. |
| Toroyan et al. (135) | 2004 | UK | journal article | reflection on a PE | health | trial | Value of process evaluation | This paper outlines the process evaluation that was conducted alongside the first RCT of day care in the United Kingdom. |
| Tuchman (136) | 2008 | USA | journal article | development of a PE approach | health | pilot/feasibility study | Use of a method / theory in process evaluation | This article presents an exemplar of a model-guided process evaluation. |
| Turner et al. (137) | 2006 | Australia | journal article | development of a PE approach | health | not specified | Process evaluation approach / framework / guidance | This paper describes the development and implementation of an electronic, web-based application to simplify data collection for this research process. |
| Viadro et al. (138) | 1997 | USA | journal article | reflection on a PE | health | quasi-experimental | Methodological / operational / ethical issues | In this paper, we discuss the steps taken to develop a process evaluation plan for an ongoing eight-year, community-based breast cancer screening program (NC-BCSP) for African American women in five rural North Carolina counties. |
| Wells et al. (139) | 2012 | UK | journal article | empirical research | health | trial | Methodological / operational / ethical issues | However, the diverse ways in which context may challenge the central tenets of the RCT, and the degree to which this information is known to researchers or subsequently reported, has received much less attention. In this paper, we explore these issues by focusing on seven RCTs of interventions varying in type and degree of complexity, and across diverse contexts. |
| Wickizer et al. (140) | 1993 | USA | journal article | development of a PE approach | health | standalone PE | Process evaluation approach / framework / guidance | This paper describes an approach developed to analyze community activation for health promotion and presents data on selected measures of activation collected in 28 communities in the western United States as part of the Community Health Promotion Grant Program evaluation.1 |
| Wierenga et al. (141) | 2013 | Netherlands | journal article | systematic review | health | trial | Review | The aim of this review was therefore to: (1) further our understanding of the quality of process evaluations alongside effect evaluations for worksite health promotion programs (WHPPs), (2) identify barriers/facilitators affecting implementation, and (3) explore the relationship between effectiveness and the implementation process. |
| Wight and Obasi (142) | 2003 | UK | book chapter | discussion and recommendations on broad topic of process evaluation | health | trial | Methodological / operational / ethical issues | This chapter discusses four key intervention factors that can be of critical importance in interpreting outcome evaluations. These factors are: (1) the extent and quality of intervention delivery; (2) the mechanism; (3) the context; and, (4) the response of the target group. Finally, the chapter considers some key problems with process evaluations and how process and outcome data can be integrated. |
| Wilson et al. (143) | 2009 | USA | journal article | reflection on a PE | health | trial | Value of process evaluation | The purpose of this study was to demonstrate how formative program process evaluation was used to improve dose and fidelity of implementation, as well as reach of the intervention into the target population, in the "Active by Choice Today" (ACT) randomized school-based trial from years 1 to 3 of implementation. |
| Windsor et al. (144) | 2000 | USA | journal article | development of a PE approach | health | trial | Process evaluation approach / framework / guidance | To describe and apply a process evaluation model (PEM) for patient education programs for pregnant smokers. |
| Yamada et al. (145) | 2010 | Canada | journal article | development of a PE approach | health | not specified | Process evaluation approach / framework / guidance | To examine the content validity of the Process Evaluation Checklist (PEC), a newly developed measure to assess the fidelity of the EPIC intervention |
| Yeary et al. (146) | 2012 | USA | journal article | systematic review | health | not specified | Review | Thus, a systematic review of the utilization of process evaluation in church-based health programs was conducted. |
| Zbukvic et al. (147) | 2020 | Australia | journal article | systematic review | health | trial | Review | This paper reviews process evaluations associated with multilevel suicide prevention research trials. |

**References**

1. Aarestrup AK, Jørgensen TS, Due P, Krølner R. A six-step protocol to systematic process evaluation of multicomponent cluster-randomised health promoting interventions illustrated by the Boost study. Evaluation and Program Planning. 2014;46:58-71.

2. Abildgaard JS, Saksvik PØ, Nielsen K. How to Measure the Intervention Process? An Assessment of Qualitative and Quantitative Approaches to Data Collection in the Process Evaluation of Organizational Interventions. Frontiers in Psychology. 2016;7(1380).

3. Abraham C, Johnson BT, Bruin dM, Luszczynska A. Enhancing reporting of behavior change intervention evaluations. Journal of Acquired Immune Deficiency Syndromes. 2014;66(Supplement 3):S293-S9.

4. Alia KA, Wilson DK, McDaniel T, St. George SM, Kitzman-Ulrich H, Smith K, et al. Development of an innovative process evaluation approach for the Families Improving Together (FIT) for weight loss trial in African American adolescents. Evaluation and Program Planning. 2015;49(Supplement C):106-16.

5. Audrey S, Holliday J, Parry-Langdon N, Campbell R. Meeting the Challenges of Implementing Process Evaluation within Randomized Controlled Trials: The Example of ASSIST (A Stop Smoking in Schools Trial). Health Education Research. 2006;21(3):366-77.

6. Bakker FC, Persoon A, Schoon Y, Olde Rikkert MGM. Uniform presentation of process evaluation results facilitates the evaluation of complex interventions: development of a graph: Presenting process evaluation's results. Journal of Evaluation in Clinical Practice. 2015;21(1):97-102.

7. Bakker FC, Persoon A, Reelick MF, van Munster BC, Hulscher M, Olde Rikkert M. Evidence from multicomponent interventions: value of process evaluations. J Am Geriatr Soc. 2013;61(5):844-5.

8. Baranowski T, Stables G. Process evaluations of the 5-a-Day projects. Health Education and Behavior. 2000;27(2):157-66.

9. Biron CaK-M, M. Process evaluation for organizational stress and well-being interventions: Implications for theory, method, and practice. International Journal of Stress Management. 2014;21(1):85-111.

10. Boeije HR, Drabble SJ, O'Cathain A. Methodological challenges of mixed methods intervention evaluations. Methodology: European Journal of Research Methods for the Behavioral and Social Sciences. 2015;11(4):119-25.

11. Brand SL, Quinn C, Pearson M, Lennox C, Owens C, Kirkpatrick T, et al. Building programme theory to develop more adaptable and scalable complex interventions: realist formative process evaluation prior to full trial. Evaluation. 2019;25(2):149-70.

12. Branscum P, Hayes L. The utilization of process evaluations in childhood obesity intervention research: A review of reviews. International Journal of Child Health and Nutrition. 2013;2(4):270-80.

13. Britton A, Thorogood M, Coombes Y, Lewando-Hundt G. Search for evidence of effective health promotion: Quantitative outcome evaluation with qualitative process evaluation is best. BMJ: British Medical Journal. 1998;316(7132):703.

14. Buckley L, Sheehan M. A process evaluation of an injury prevention school-based programme for adolescents. Health education research. 2009;24(3):507-19.

15. Bunce AE, Gold R, Davis JV, McMullen CK, Jaworski V, Mercer M, et al. Ethnographic process evaluation in primary care: explaining the complexity of implementation. BMC Health Services Research. 2014;14(1):1-10.

16. Butterfoss FD. Process Evaluation for Community Participation. Annual review of public health. 2006. 27(1):323-40.

17. Byng R, Norman I, Redfern S. Using Realistic Evaluation to Evaluate a Practice-level Intervention to Improve Primary Healthcare for Patients with Long-term Mental Illness. Evaluation. 2005;11(1):69-93.

18. Byng R, Norman I, Redfern S, Jones R. Exposing the key functions of a complex intervention for shared care in mental health: case study of a process evaluation. BMC health services research. 2008;8(1):274-.

19. Chandler J. Application of simplified Complexity Theory concepts for healthcare social systems to explain the implementation of evidence into practice. Journal of advanced nursing. 2016;72(2):461-80.

20. Cheng KK, Metcalfe A. Qualitative methods and process evaluation in clinical trials context: Where to head to? : SAGE Publications Sage CA: Los Angeles, CA; 2018.

21. Chrisman NJ, Senturia K, Tang G, Gheisar B. Qualitative process evaluation of urban community work: a preliminary view. Health education & behavior. 2002;29(2):232-48.

22. Cornwall A, Aghajanian A. How to find out what’s really going on: understanding impact through participatory process evaluation. World Development. 2017;99:173-85.

23. Crutzen R. Using Google Analytics as a process evaluation method for Internet-delivered interventions: an example on sexual health. Health promotion international. 2012;28(1):36-42.

24. Cunningham LE. The value of process evaluation in a community-based cancer control program. Evaluation and program planning. 2000;23(1):13-25.

25. De Silva MJ, Breuer E, Lee L, Asher L, Chowdhary N, Lund C, et al. Theory of Change: a theory-driven approach to enhance the Medical Research Council's framework for complex interventions. Trials. 2014;15(1):267-.

26. Diaz T, Guenther T, Oliphant NP, Muñiz M, i CCMSioetg. A proposed model to conduct process and outcome evaluations and implementation research of child health programs in Africa using integrated community case management as an example. Journal of global health. 2014;4(2):020409.

27. Ekambareshwar M, Ekambareshwar S, Mihrshahi S, Wen LM, Baur LA, Laws R, et al. Process evaluations of early childhood obesity prevention interventions delivered via telephone or text messages: a systematic review. International Journal of Behavioral Nutrition and Physical Activity. 2021;18(1):1-25.

28. Ellard DR, Parsons S. Process evaluation: understanding how and why interventions work. In: Thorogood M, Coombes Y, editors. Evaluating health promotion: practice and methods. 3rd ed. Oxford: Oxford University Press; 2010.

29. Ellard DR, Taylor SJC, Parsons S, Thorogood M. The OPERA trial: A protocol for the process evaluation of a randomised trial of an exercise intervention for older people in residential and nursing accommodation. Trials. 2011;12(1):28-.

30. Evans R, Scourfield J, Murphy S. Pragmatic, formative process evaluations of complex interventions and why we need more of them. Journal of epidemiology and community health. 2015;69(10):925-6.

31. Ferm L, Rasmussen CDN, Jørgensen MB. Operationalizing a model to quantify implementation of a multi-component intervention in a stepped-wedge trial. Implementation Science. 2018;13(1):26.

32. Francis JJ, Eccles MP, Johnston M, Whitty P, Grimshaw JM, Kaner EF, et al. Explaining the effects of an intervention designed to promote evidence-based diabetes care: a theory-based process evaluation of a pragmatic cluster randomised controlled trial. Implementation Science. 2008;3(1):50.

33. Franzen S, Morrel-Samuels S, Reischl TM, Zimmerman MA. Using Process Evaluation to Strengthen Intergenerational Partnerships in the Youth Empowerment Solutions Program. Journal of Prevention & Intervention in the Community. 2009;37(4):289-301.

34. French C, Pinnock H, Forbes G, Skene I, Taylor SJ. Process evaluation within pragmatic randomised controlled trials: what is it, why is it done, and can we find it?—a systematic review. Trials. 2020;21(1):1-16.

35. Frost J, Wingham J, Britten N, Greaves C, Abraham C, Warren FC, et al. The value of social practice theory for implementation science: learning from a theory-based mixed methods process evaluation of a randomised controlled trial. BMC Medical Research Methodology. 2020;20(1):1-14.

36. Gale RC, Wu J, Erhardt T, Bounthavong M, Reardon CM, Damschroder LJ, et al. Comparison of rapid vs in-depth qualitative analytic methods from a process evaluation of academic detailing in the Veterans Health Administration. Implementation Science. 2019;14(1):1-12.

37. Gensby U, Braathen TN, Jensen C, Eftedal M. Designing a process evaluation to examine mechanisms of change in return to work outcomes following participation in occupational rehabilitation: a theory-driven and interactive research approach. International Journal of Disability Management. 2018;13.

38. Gobat NH, Littlecott H, Williams A, McEwan K, Stanton H, Robling M, et al. Developing whole-school mental health and wellbeing intervention through pragmatic formative process evaluation: A case-study of innovative local practice within the School Health Research Network. 2020.

39. Grant A, Dreischulte T, Treweek S, Guthrie B. Study protocol of a mixed-methods evaluation of a cluster randomized trial to improve the safety of NSAID and antiplatelet prescribing: data-driven quality improvement in primary care. Trials. 2012;13(1):154.

40. Grant A, Treweek S, Dreischulte T, Foy R. Process evaluations for cluster-randomised trials of complex interventions: a proposed framework for design and reporting. Trials. 2013;14(1):15.

41. Grant A, Treweek S, Wells M. Why is so much clinical research ignored and what do we do about it? British Journal of Hospital Medicine. 2016.

42. Grant A, Bugge C, Wells M. Designing process evaluations using case study to explore the context of complex interventions evaluated in trials. Trials. 2020;21(1):1-10.

43. Gray CS, Shaw J. From summative to developmental: incorporating design-thinking into evaluations of complex interventions. Journal of Integrated Care. 2019.

44. Griffin TL, Pallan MJ, Clarke JL, Lancashire ER, Lyon A, Parry JM, et al. Process evaluation design in a cluster randomised controlled childhood obesity prevention trial: the WAVES study. International Journal of Behavioral Nutrition and Physical Activity. 2014;11(1):112.

45. Griffin T, Clarke J, Lancashire E, Pallan M, Adab P. Process evaluation results of a cluster randomised controlled childhood obesity prevention trial: the WAVES study. BMC public health. 2017;17(1):681.

46. Harachi TW, Abbott RD, Catalano RF, Haggerty KP, Fleming CB. Opening the black box: using process evaluation measures to assess implementation and theory building. Am J Community Psychol. 1999;27(5):711.

47. Harvey L, Jan S. Process evaluations for large clinical trials involving complex interventions. Spinal cord. 2017;55(11):963-.

48. Hatcher AM, Bonell CP. High time to unpack the 'how' and 'why' of adherence interventions. AIDS (London). 2016;30(8):1301-3.

49. Havermans BM, Schelvis RM, Boot CR, Brouwers EP, Anema JR, van der Beek AJ. Process variables in organizational stress management intervention evaluation research: a systematic review. Scandinavian journal of work, environment & health. 2016;42(5):371-81.

50. Hawe P, Shiell A, Riley T. Complex interventions: how "out of control" can a randomised controlled trial be? BMJ: British Medical Journal. 2004;328(7455):1561-3.

51. Haynes A, Brennan S, Carter S, O'Connor D, Schneider CH. Protocol for the process evaluation of a complex intervention designed to increase the use of research in health policy and program organisations (the SPIRIT study). Implementation science : IS. 2014;9(1):113.

52. Haynes A, Brennan S, Redman S, Williamson A, Gallego G, Butow P. Figuring out fidelity: a worked example of the methods used to identify, critique and revise the essential elements of a contextualised intervention in health policy agencies. Implementation Science. 2016;11(1):23.

53. Helitzer D, Yoon SJ, Wallerstein N, Garcia‐Velarde LDy. The role of process evaluation in the training of facilitators for an adolescent health education program. Journal of School Health. 2000;70(4):141-7.

54. Hickey G, McGilloway S, Furlong M, Leckey Y, Bywater T. Understanding the implementation and effectiveness of a group-based early parenting intervention: a process evaluation protocol. BMC health services research. 2016;16(1):490.

55. Hulscher MEJL, Laurant MGH, Grol RPTM. Process evaluation on quality improvement interventions. Quality & safety in health care. 2003;12(1):40-6.

56. Humphrey N, Lendrum A, Ashworth E, Frearson K, Buck R, Kerr K. Implementation and process evaluation (IPE) for interventions in educational settings: An introductory handbook. London, UK: Education Endowment Foundation; 2016.

57. Humphrey N, Lendrum A, Ashworth E, Frearson K, Buck R, Kerr K. Implementation and process evaluation (IPE) for interventions in educational settings: A synthesis of the literature. London, UK: Education Endowment Foundation; 2016.

58. Irvine L, Falconer DW, Jones C, Ricketts IW, Williams B. Can text messages reach the parts other process measures cannot reach: an evaluation of a behavior change intervention delivered by mobile phone? PloS one. 2012;7(12):e52621.

59. Jansen YJFM, Foets MME, de Bont AA. The contribution of qualitative research to the development of tailor-made community-based interventions in primary care: a review. European Journal of Public Health. 2009;20(2):220-6.

60. Kelley SD, Van Horn M, DeMaso DR. Using process evaluation to describe a hospital-based clinic for children coping with medical stressors. Journal of pediatric psychology. 2001;26(7):407-15.

61. Kostamo K. Using the critical incident technique for qualitative process evaluation of interventions: The example of the "Let's Move It" trial. Social science & medicine (1982). 2019;232.

62. Koutsouris G, Norwich B, Stebbing J. The significance of a process evaluation in interpreting the validity of an RCT evaluation of a complex teaching intervention: the case of Integrated Group Reading (IGR) as a targeted intervention for delayed Year 2 and 3 pupils. Cambridge Journal of Education. 2019;49(1):15-33.

63. Lee BK, Lockett D, Edwards N. Gauging alignments: an ethnographyically informed method for process evaluation in a community-based intervention. 2011.

64. Lee H, Contento IR, Koch P. Using a systematic conceptual model for a process evaluation of a middle school obesity risk-reduction nutrition curriculum intervention: choice, control & change. Journal of nutrition education and behavior. 2013;45(2):126-36.

65. Leeming D, Marshall J, Locke A. Understanding process and context in breastfeeding support interventions: The potential of qualitative research Understanding process in breastfeeding support. Maternal and child nutrition. 2017;13(4).

66. Legrand K, Minary L, Briançon S. Exploration of the experiences, practices and needs of health promotion professionals when evaluating their interventions and programmes. Evaluation and program planning. 2018;70:67-72.

67. Leontjevas R, Gerritsen DL, Koopmans RTCM, Smalbrugge M, Vernooij-Dassen MJFJ. Process evaluation to explore internal and external validity of the "Act in Case of Depression" care program in nursing homes. Journal of the American Medical Directors Association. 2012;13(5):488.e1-.e8.

68. Limbani F. Process evaluation in the field: global learnings from seven implementation research hypertension projects in low-and middle-income countries. BMC public health. 2019;19(1).

69. Linnan L, Steckler A. Process evaluation for public health interventions and research: an overview. In: Steckler A, Linnan L, editors. Process evaluation for public health interventions and research. San Francisco Jossey-Bass; 2002.

70. Liu H, Muhunthan J, Hayek A, Hackett M, Laba T-L, Peiris D, et al. Examining the use of process evaluations of randomised controlled trials of complex interventions addressing chronic disease in primary health care—a systematic review protocol. Systematic Reviews. 2016;5(1):138.

71. Liu H, Mohammed A, Shanthosh J, Laba T-L, Hackett ML, Peiris D, et al. Process evaluations of primary care interventions addressing chronic disease: a systematic review. BMJ open. 2019;9(8):e025127.

72. Lorencatto F, Gould NJ, McIntyre SA, During C, Bird J. A multidimensional approach to assessing intervention fidelity in a process evaluation of audit and feedback interventions to reduce unnecessary blood transfusions: a study protocol. Implementation science : IS. 2016;11(1):163.

73. Lytle LA, Davidann BZ, Bachman K, Edmundson EW, Johnson CC, Reeds JN, et al. CATCH: Challenges of conducting process evaluation in a multicenter trial. Health education quarterly. 1994;21(1_suppl):S129-S41.

74. Maar MA, Yeates K, Perkins N, Boesch L, Hua-Stewart D, Liu P, et al. A Framework for the Study of Complex mHealth Interventions in Diverse Cultural Settings. JMIR mHealth and uHealth. 2017;5(4).

75. Manchaiah V, Danermark B, Rönnberg J, Lunner T, Linköpings u, Institutionen för beteendevetenskap och l, et al. Importance of “Process Evaluation” in Audiological Rehabilitation: Examples from Studies on Hearing Impairment. International Journal of Otolaryngology. 2014;2014:1-7.

76. Masterson Algar P. Advancing process evaluation research within the field of neurological rehabilitation. [dissertation on the internet]. Bangor: Prifysgol Bangor University; 2016 [cited 15 Mar 2022]. Available from: <https://research.bangor.ac.uk/portal/en/theses/advancing-process-evaluation-oresearch-within-the-filed-of-neurological-rehabilitation(7f9921d6-245d-4697-8617-1cddbb43a85f).html>

77. Masterson-Algar P, Burton CR, Rycroft-Malone J. Process evaluations in neurological rehabilitation: a mixed-evidence systematic review and recommendations for future research. BMJ Open. 2016;6(11):e013002.

78. Masterson-Algar P, Burton CR, Rycroft-Malone J. The generation of consensus guidelines for carrying out process evaluations in rehabilitation research. BMC Medical Research Methodology. 2018;18(1):180.

79. May CR, Mair FS, Dowrick CF, Finch TL. Process evaluation for complex interventions in primary care: understanding trials using the normalization process model. BMC family practice. 2007;8(1):42.

80. May CR, Cummings A, Girling M, Bracher M, Mair FS, May CM, et al. Using Normalization Process Theory in feasibility studies and process evaluations of complex healthcare interventions: a systematic review. Implementation Science. 2018;13(1):80.

81. Mbuya MNN. Theory-Driven Process Evaluation of the SHINE Trial Using a Program Impact Pathway Approach. Clinical infectious diseases. 2015;61(suppl 7):S752-S8.

82. McGill E, Marks D, Er V, Penney T, Petticrew M, Egan M. Qualitative process evaluation from a complex systems perspective: A systematic review and framework for public health evaluators. PLoS medicine. 2020;17(11):e1003368.

83. McGraw SA, Stone EJ, Osganian SK, Elder JP, Perry CL, Johnson CC, et al. Design of process evaluation within the Child and Adolescent Trial for Cardiovascular Health (CATCH). Health Education Quarterly. 1994;21(1_suppl):S5-S26.

84. McIntyre SA, Francis JJ, Gould NJ, Lorencatto F. The use of theory in process evaluations conducted alongside randomized trials of implementation interventions: A systematic review. Translational behavioral medicine. 2020;10(1):168-78.

85. Moore G. Developing a mixed methods framework for process evaluations of complex interventions: the case of the National Exercise Referral Scheme policy trial in Wales. [dissertation on the internet] Cardiff: University of Cardiff; 2010 [cited 15 Mar 2022] Available from: https://orca.cardiff.ac.uk/55051/

86. Moore G, Audrey S, Barker M, Bond L, Bonell C, Hardeman W, et al. Process evaluation of complex interventions: UK Medical Research Council (MRC) guidance. London: MRC Population Health Science Network; 2014.

87. Moore G, Audrey S, Barker M, Bond L, Bonell C, Cooper C, et al. Process evaluation in complex public health intervention studies: the need for guidance. Journal of epidemiology and community health. 2014;68(2):101-2.

88. Moore GF, Audrey S, Barker M, Bond L, Bonell C, Hardeman W, et al. Process evaluation of complex interventions: Medical Research Council guidance. British Medical Journal. 2015;350:h1258.

89. Morgan-Trimmer S. Improving process evaluations of health behavior interventions: learning from the social sciences. Evaluation & the health professions. 2015;38(3):295-314.

90. Morgan-Trimmer S, Wood F. Ethnographic methods for process evaluations of complex health behaviour interventions. Trials. 2016;17(1):232.

91. Morrel-Samuels S, Rupp LA, Eisman AB, Miller AL, Stoddard SA, Franzen SP, et al. Measuring the implementation of youth empowerment solutions. Health promotion practice. 2018;19(4):581-9.

92. Munodawafa M. Process evaluations of task sharing interventions for perinatal depression in low and middle income countries (LMIC): a systematic review and qualitative meta-synthesis. BMC Health Services Research. 2018;18(1):205.

93. Munro A, Bloor M. Process evaluation: the new miracle ingredient in public health research? Qualitative Research. 2010;10(6):699-713.

94. Murdoch J. Process evaluation for complex interventions in health services research: analysing context, text trajectories and disruptions. BMC health services research. 2016;16(1):407.

95. Murta SG, Sanderson K, Oldenburg B. Process evaluation in occupational stress management programs: a systematic review. American Journal of Health Promotion 2007;21(4):248.

96. Murtagh M, Thomson R, May C, Rapley T, Heaven B, Graham R, et al. Qualitative methods in a randomised controlled trial: the role of an integrated qualitative process evaluation in providing evidence to discontinue the intervention in one arm of a trial of a decision support tool. Quality and Safety in Health care. 2007;16(3):224-9.

97. Nagy MC, Johnson RE, Vanderpool RC, Fouad MN, Dignan M, Wynn TA, et al. Process evaluation in action: lessons learned from Alabama REACH 2010. Journal of Health Disparities Research and Practice. 2008;2(1):6.

98. Nielsen K, Karina N, Johan Simonsen A. Organizational interventions: A research-based framework for the evaluation of both process and effects. Work and stress. 2013;27(3):278.

99. Nielsen K, Randall R. Opening the black box: Presenting a model for evaluating organizational-level interventions. European Journal of Work and Organizational Psychology. 2013;22(5):601-17.

100. Nielsen JN, Olney DK, Ouedraogo M, Pedehombga A, Rouamba H, Yago‐Wienne F. Process evaluation improves delivery of a nutrition‐sensitive agriculture programme in Burkina Faso. Maternal & child nutrition. 2018;14(3):e12573.

101. Oakley A. Evaluating Processes A Case Study of a Randomized Controlled Trial of Sex Education. Evaluation (London, England 1995). 2004;10(4):440-62.

102. Oakley A, Strange V, Bonell C, Allen E, Stephenson J. Process evaluation in randomised controlled trials of complex interventions. BMJ (Clinical research ed). 2006;332(7538):413-6.

103. O’Cathain A, Thomas KJ, Drabble SJ, Rudolph A, Goode J, Hewison J. Maximising the value of combining qualitative research and randomised controlled trials in health research: the QUAlitative Research in Trials (QUART) study–a mixed methods study. Health Technology Assessment. 2014;18(38).

104. Odendaal WA, Marais S, Munro S, van Niekerk A. When the trivial becomes meaningful: Reflections on a process evaluation of a home visitation programme in South Africa. Evaluation and Program Planning. 2008;31(2):209-16.

105. Ottoson JM, Patterson I. Contextual influences on learning application in practice: An extended role for process evaluation. Evaluation & the health professions. 2000;23(2):194-211.

106. Palmer VJ, Piper D, Richard L, Furler J, Herrman H. Balancing Opposing Forces—A Nested Process Evaluation Study Protocol for a Stepped Wedge Designed Cluster Randomized Controlled Trial of an Experience Based Codesign Intervention The CORE Study. International journal of qualitative methods. 2016;15(1):160940691667221.

107. Parker AM. Process evaluation and the development of behavioural interventions to improve psychological distress among survivors of critical illness. Thorax. 2019;74(1).

108. Parrott A, Carman JG. Scaling Up Programs: Reflections on the Importance of Process Evaluation. Canadian Journal of Program Evaluation. 2019;34(1).

109. Platt S, Gnich W, Rankin D, Ritchie D, Truman J, Backett-Milburn K. Applying process evaluation: Learning from two research projects. 2009. In: Thorogood M, Coombes Y, editors. Evaluating Health Promotion: Practice and Methods. Oxford Scholarship Online.

110. Raine R, Fitzpatrick R, Barratt H, Bevan G, Black N, Boaden R, et al. Challenges, solutions and future directions in the evaluation of service innovations in health care and public health. Health Services and Delivery Research. 2016;4(16).

111. Ramsay CR, Thomas RE, Croal BL, Grimshaw JM, Eccles MP. Using the theory of planned behaviour as a process evaluation tool in randomised trials of knowledge translation strategies: a case study from UK primary care. Implementation Science. 2010;5(1):71.

112. Redmond N, Harker L, Bamps Y, Flemming SSC, Perryman JP, Thompson NJ, et al. Implementation of a Web-Based Organ Donation Educational Intervention: Development and Use of a Refined Process Evaluation Model. Journal of medical Internet research. 2017;19(11):e396.

113. Renger R, Foltysova J. Deliberation-derived process (DDP) evaluation. Evaluation Journal of Australasia. 2013;13(2):9.

114. Reynolds J, DiLiberto D, Mangham-Jefferies L, Ansah E, Lal S, Mbakilwa H, et al. The practice of 'doing' evaluation: lessons learned from nine complex intervention trials in action. Implementation Science. 2014;9(1):75.

115. Ribeiro DC. Process evaluation of complex interventions tested in randomised controlled trials in musculoskeletal disorders: a systematic review protocol. BMJ open. 2019;9(5).

116. Riley T, Hawe P, Shiell A. Contested ground: how should qualitative evidence inform the conduct of a community intervention trial? Journal of health services research & policy. 2005;10(2):103-10.

117. Roberts-Gray C. Structuring Process Evaluation to Forecast Use and Sustainability of an Intervention: Theory and Data From the Efficacy Trial for. Health education & behavior. 2017;44(4):559-69.

118. Roe K, Roe K. Dialogue boxes: a tool for collaborative process evaluation. Health promotion practice. 2004;5(2):138-50.

119. Rycroft-Malone J. A realist process evaluation within the Facilitating Implementation of Research Evidence (FIRE) cluster randomised controlled international trial: an exemplar. Implementation science : IS. 2018;13(1).

120. Saarijärvi M, Wallin L, Bratt E-L. Process evaluation of complex cardiovascular interventions: How to interpret the results of my trial? European Journal of Cardiovascular Nursing. 2020;19(3):269-74.

121. Saunders RP, Evans MH, Joshi P. Developing a process-evaluation plan for assessing health promotion program implementation: a how-to guide. Health promotion practice. 2005;6(2):134-47.

122. Scantlebury A, Cockayne S, Fairhurst C, Rodgers S, Torgerson D, Hewitt C, et al. Qualitative research to inform hypothesis testing for fidelity-based sub-group analysis in clinical trials: lessons learnt from the process evaluation of a multifaceted podiatry intervention for falls prevention. Trials. 2020;21(1):348.

123. Scott SD, Rotter T, Hartling L, Chambers T, Bannar-Martin KH. A protocol for a systematic review of the use of process evaluations in knowledge translation research. Systematic reviews. 2014;3(1):149.

124. Scott SD, Rotter T, Flynn R, Brooks HM, Plesuk T, Bannar-Martin KH, et al. Systematic review of the use of process evaluations in knowledge translation research. Systematic reviews. 2019;8(1):266.

125. Segrott J, Murphy S, Rothwell H, Scourfield J, Foxcroft D, Gillespie D, et al. An application of extended normalisation process theory in a randomised controlled trial of a complex social intervention: process evaluation of the strengthening families Programme (10–14) in Wales, UK. SSM-population health. 2017;3:255-65.

126. Sharma S, Adetoro OO, Vidler M, Drebit S, Payne BA, Akeju DO, et al. A process evaluation plan for assessing a complex community-based maternal health intervention in Ogun State, Nigeria. BMC health services research. 2017;17(1):238.

127. Shepherd HL, Geerligs L, Butow P, Masya L, Shaw J, Price MA, et al. The elusive search for success: defining and measuring implementation outcomes in a real-world hospital trial. Frontiers in public health. 2019;7:293.

128. Siddiqui N, Gorard S, See BH. The importance of process evaluation for randomised control trials in education. Educational Research. 2018;60(3):357-70.

129. Simuyemba MC, Ndlovu O, Moyo F, Kashinka E, Chompola A, Sinyangwe A, et al. Real-time evaluation pros and cons: Lessons from the Gavi Full Country Evaluation in Zambia. Evaluation. 2020:1356389019901314.

130. Stappers N, Van Kann D, Jansen M, Kremers S, De Vries N, Bekker M. The role of context in evaluation studies: Lessons from a process evaluation of integrating health in urban reconstruction. Environmental Impact Assessment Review. 2020;82:106365.

131. Strange V, Allen E, Oakley A, Bonell C, Johnson A, Stephenson J, et al. Integrating process with outcome data in a randomized controlled trial of sex education. Evaluation. 2006;12(3):330-52.

132. Tolma EL, Cheney MK, Troup P, Hann N. Designing the process evaluation for the collaborative planning of a local turning point partnership. Health promotion practice. 2009;10(4):537-48.

133. Tolma EL, Cheney MK, Chrislip DD, Blankenship D, Troup P, Hann N. A Systematic Approach to Process Evaluation in the Central Oklahoma Turning Point (COTP) Partnership. American Journal of Health Education. 2011;42(3):130-41.

134. Tonkin-Crine S, Anthierens S, Hood K, Yardley L, Cals JWL. Discrepancies between qualitative and quantitative evaluation of randomised controlled trial results: achieving clarity through mixed methods triangulation. Implementation science : IS. 2016;11(1).

135. Toroyan T, Oakley A, Laing G, Roberts I, Mugford M, Turner J. The impact of day care on socially disadvantaged families: an example of the use of process evaluation within a randomized controlled trial. Child: care, health and development. 2004;30(6):691-8.

136. Tuchman E. A model-guided process evaluation: Office-based prescribing and pharmacy dispensing of methadone. Evaluation and Program Planning. 2008;31(4):376-81.

137. Turner C, Yorkston E, Hart K, Drew L, McClure R. Simplifying data collection for process evaluation of community coalition activities-an electronic web-based application. Health promotion journal of Australia. 2006;17(1):48-53.

138. Viadro CI, Earp JAL, Altpeter M. Designing a process evaluation for a comprehensive breast cancer screening intervention: Challenges and opportunities. Evaluation and Program Planning. 1997;20(3):237-49.

139. Wells M, Williams B, Treweek S, Coyle J, Taylor J. Intervention description is not enough: evidence from an in-depth multiple case study on the untold role and impact of context in randomised controlled trials of seven complex interventions. Trials. 2012;13(1):95-.

140. Wickizer TM, Von Korff M, Cheadle A, Maeser J, Wagner EH, Pearson D, et al. Activating communities for health promotion: a process evaluation method. American Journal of Public Health. 1993;83(4):561-7.

141. Wierenga D, Engbers LH, Van Empelen P, Duijts S, Hildebrandt VH, Van Mechelen W. What is actually measured in process evaluations for worksite health promotion programs: a systematic review. BMC Public Health. 2013;13(1):1190.

142. Wight D, Obasi A. Unpacking the ‘black box’: the importance of process data to explain outcomes. In: Stephenson JM, Bonell C, Imrie J, editors. Effective sexual health interventions : issues in experimental evaluation. Oxford: Oxford University Press; 2003.

143. Wilson DK, Griffin S, Saunders RP, Kitzman-Ulrich H, Meyers DC. Using process evaluation for program improvement in dose, fidelity and reach: the ACT trial experience. The international journal of behavioral nutrition and physical activity. 2009;6(1):79.

144. Windsor RA, Whiteside HP, Solomon LJ, Prows SL, Donatelle RJ, Cinciripini PM, et al. A process evaluation model for patient education programs for pregnant smokers. Tobacco Control. 2000;9(suppl 3):iii29-iii35.

145. Yamada J, Stevens B, Sidani S, Watt‐Watson J, De Silva N. Content validity of a process evaluation checklist to measure intervention implementation fidelity of the EPIC intervention. Worldviews on Evidence‐Based Nursing. 2010;7(3):158-64.

146. Yeary KH, Klos LA, Linnan L. The examination of process evaluation use in church-based health interventions: a systematic review. Health Promotion Practice. 2012;13(4):524-34.

147. Zbukvic IC, Mok K, McGillivray L, Chen NA, Shand FL, Torok MH. Understanding the process of multilevel suicide prevention research trials. Evaluation and program planning. 2020;82:101850.
